# Benefits of an anti-inflammatory diet compared to a low-residue diet during concurrent chemoradiation therapy for patients with locally advanced cervical cancer: a randomized clinical trial

**DOI:** 10.3389/fnut.2026.1835417

**Published:** 2026-07-13

**Authors:** Julissa Luvián-Morales, Ariadna Alejandra Rueda-Escalona, Merari Delgadillo-González, Lucely del Carmen Cetina-Pérez, Diana Carolina Villalpando-Sánchez, Aaron Mauricio Gómez-Jiménez, Marcos Isaac Rodríguez-Moreno, Oscar Medina-Contreras, Denisse Castro-Eguiluz

**Affiliations:** 1 MICAELA Program, Instituto Nacional de Cancerología, Mexico City, Mexico; 2 Department of Clinical Research, Instituto Nacional de Cancerología, Mexico City, Mexico; 3 Masters in Medical Sciences Program, Universidad Anáhuac Mexico, Huixquilucan, Mexico; 4 Epidemiology, Endocrinology, and Nutrition Research Unit, Hospital Infantil de México Federico Gómez, Mexico City, Mexico; 5 Department of Mathematics, School of Sciences. Universidad Nacional Autónoma de México, Mexico City, Mexico; 6 Department of Actuary, School of Sciences, Universidad Nacional Autónoma de México, Mexico City, Mexico; 7 Secretaría de Ciencia, Humanidades, Tecnología e Innovación (SECIHTI)— Department of Clinical Research, Instituto Nacional de Cancerología, Mexico City, Mexico

**Keywords:** anti-inflammatory diet, calprotectin, cancer undernutrition, cervical cancer, inflammation, overall survival, proctopathy, treatment toxicity

## Abstract

**Background:**

Gastrointestinal (GI) toxicity frequently occurs in patients with locally advanced cervical cancer receiving concurrent chemoradiotherapy, often resulting in severe malnutrition.

**Purpose:**

This study compared the effects of an anti-inflammatory diet (AID) versus a low-residue diet (LRD) on nutritional status, toxicity, inflammation, and treatment outcomes in women with locally advanced cervical cancer (LACC) undergoing chemoradiotherapy (CRT).

**Methods:**

In this randomized controlled trial, women with LACC were assigned to either the AID or the LRD. Nutritional status, GI toxicity, inflammation, and tumor response were assessed at five time points, before, during, and after treatment.

**Results:**

A total of 83 participants completed the study: 41 in the AID group and 42 in the LRD group. The AID group consumed more anti-inflammatory nutrients, and both groups showed nutritional recovery by visit 5, as indicated by Patient-Generated Subjective Global Assessment (PG-SGA) scores and lower rates of undernutrition. Although both groups experienced peak toxicity mid-treatment, the AID group reported fewer persistent GI symptoms by the end, consistent with more stable intestinal inflammation, as evidenced by calprotectin levels. Cytokine analysis showed lower IL-1β and IL-17A levels in the AID group at the final visit. A complete response to treatment was seen in 70.7% of the AID group versus 54.8% in the LRD group (*p* = 0.133). Overall survival rates were more favorable in the AID group.

**Conclusion:**

In women with LACC receiving CRT, the anti-inflammatory diet showed a trend toward improved nutritional status, reduced inflammation, and better treatment response. These findings underscore the potential clinical benefits of incorporating personalized, nutrient-rich dietary interventions into supportive oncology care. The study was registered on ClinicalTrials.gov (ID: NCT03994055).

**Clinical Trial Registration:**

https://clinicaltrials.gov/study/NCT03994055, identifier [NCT03994055]

## 1 Introduction

Undernutrition is a frequent condition in cancer patients, affecting approximately 80% during oncological treatment, with up to 20% related mortality (1). Undernutrition in cancer is multifactorial, influenced by tumor type, cancer stage, individual patient characteristics, oncological treatments, and sociodemographic conditions (2). Tumors can manipulate energy and nutrient acquisition by releasing cytokines and tumor-derived factors, which cause systemic inflammation and acute-phase response. This response often leads to metabolic changes and a catabolic state (1).

In locally advanced cervical cancer (LACC) patients, undernutrition occurs in up to 69% of patients following standard treatment with concurrent chemoradiotherapy (CRT) followed by brachytherapy (BT) (3). CRT also impacts healthy tissues with a high rate of cell turnover, such as the gastrointestinal mucosa (4, 5). If the damage is too severe or the cells cannot activate their intrinsic repair mechanisms, apoptosis occurs, leading to intestinal inflammation. Common gastrointestinal toxicities associated with CRT include nausea, diarrhea, constipation, abdominal pain, and bloating (6). These side effects negatively affect appetite, feeding, and nutrient absorption, contributing to undernutrition (3).

Among these toxicity symptoms, diarrhea has been the most studied in clinical trials that have explored low-residue diets (low-fiber, low-fat, and lactose-free). However, these studies showed limited improvements in diarrhea severity and did not assess patients’ nutritional status (7). Weight loss following these dietary interventions indicates that low-residue diets may promote greater nutritional deterioration (8). Evidence supporting the use of low-residue diets is limited; nevertheless, they are typically recommended as standard practice during pelvic radiotherapy (RT). We believe these guidelines are inadequate, as patients following restrictive diets often consume nutrient-poor, refined, ultra-processed low-residue foods, which can lead to dysbiosis and inflammation.

Recent research on dietary interventions in healthy adults has shown that diets rich in fiber, prebiotics, and probiotics can enhance immune responses, increase microbiome diversity, and improve gut health. The addition of probiotics has positively influenced diarrhea management (9).

Furthermore, specific functional nutrients have been linked to the regulation of inflammation in various chronic diseases, many of which are associated with cancer prevention (10). Key compounds include epigallocatechin gallate (EGCG), curcumin, and resveratrol, all of which have antioxidant properties that inhibit reactive oxygen species (ROS). Omega-3 fatty acids (Omega-3 FA), resveratrol, and EGCG may also inhibit inflammatory transcription factors like NF-κB. Vitamins A and D, along with short-chain fatty acids (SCFAs) produced by gut bacteria during fiber fermentation, can prevent dendritic cell activation and inhibit Th1 and Th17 lymphocyte differentiation (11).

Due to its immune-modulatory and potent antioxidant properties, an anti-inflammatory diet could be beneficial throughout the progression and treatment of cervical cancer (CC) (9, 12). Specifically, for patients with LACC undergoing CRT, an anti-inflammatory approach may provide elements that help regulate gut inflammation, reduce toxicity, and consequently improve nutritional status, compared to traditional low-residue diets.

In the present study, we conducted a randomized controlled clinical trial to compare the effects of a low-residue diet (LRD) with an anti-inflammatory diet (AID) that is high in fiber, antioxidants, Omega-3 FA, and probiotics. The trial aimed to assess the impact of these diets on two primary outcomes: nutritional status and systemic inflammation. The secondary outcomes included gastrointestinal toxicity, local inflammatory responses, and overall clinical outcomes.

## 2 Materials and methods

This study is a randomized controlled clinical trial that received approval from the Research and Ethics Committees (approval numbers 018/023/ICI; CEI/1247/18) of the National Cancer Institute (INCan), a tertiary research hospital in Mexico City. The research was conducted at INCan from 2018 to 2024 and was registered on ClinicalTrials.gov (ID: NCT03994055; https://clinicaltrials.gov/study/NCT03994055). A sample size was calculated for both primary outcomes, change in nutritional status and change in systemic inflammation, using a formula for two proportions (proportions for undernutrition: 81.4% for LRD and 59.7% for AID), alpha of 5%, and a power of 80%; the largest sample size was 130 patients. The sample size was calculated based on previous reports of undernutrition (3).

### 2.1 Subjects

The study included patients with LACC at stages IB3 to IVA according to the FIGO 2018 classification. Eligible participants were aged 18 years or older with a confirmed diagnosis of CC, as determined by biopsy (histological examination included adenosquamous, squamous, adenocarcinoma, or villous cells). All participants had undergone a computed tomography scan. Candidates eligible for inclusion were required to receive CRT as their first-line treatment, maintain a good functional status (ECOG score of 0-2), and be able to understand and sign an informed consent to participate in the study. Additionally, adequate organ function, confirmed through biochemical tests, was necessary. Any existing comorbidities needed to be appropriately managed.

Individuals who refused to participate, patients receiving nutritional supplements, those involved in other clinical studies, patients with fistulae, and individuals diagnosed with a second malignancy were excluded from the study.

### 2.2 Cancer treatment

All participants underwent CRT followed by BT. Chemotherapy (CT) consisted on cisplatin at a dosage of 40 mg/m^2^ or gemcitabine at 300 mg/m^2^, both administered weekly for 6 weeks. External three-dimensional radiotherapy (RT) was administered to patients using four standard pelvic field points, with a total dose of 50.4 Gy delivered in 28 fractions of 1.8 Gy each, 5 days a week. After completing CRT, low-dose or high-dose BT was applied to the intracavitary area.

### 2.3 Randomization and ethical considerations

Participants were randomly assigned to the AID or LRD group according to a pre-established list on the website randomization.org. The entire study population was divided into three blocks: 40% without comorbidities, 30% with comorbidities, and 30% with renal deterioration, based on INCan comorbidity statistics. Eligible candidates were invited to participate; those who agreed signed informed consent forms.

When a participant agreed to join the study, the nutritionist in charge sent an email to another collaborator who was not involved in participant assessments, and the group assignment was provided according to the randomization list. Allocation concealment was not possible because of the nature of the intervention.

### 2.4 Study setting and design

**FIGURE 1:**
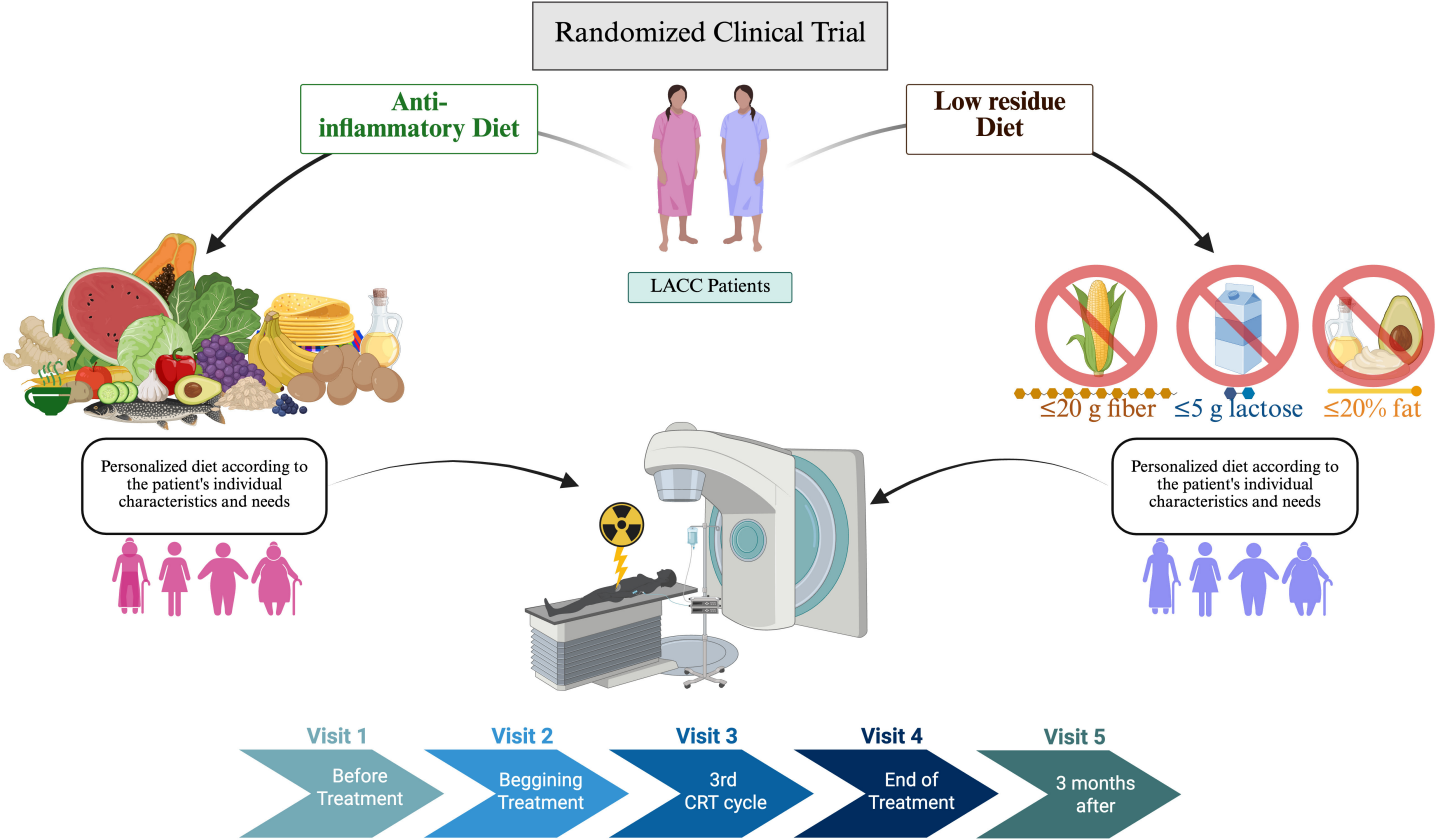
Study design. LACC patients were randomized to consume AID or LRD. Each participant received a dietary guide tailored to their individual energy and protein requirements. The AID consisted of foods such as fruits, vegetables, fish, yogurt, whole cereals, corn tortilla, chia seeds, nuts, green tea, turmeric, and ginger. The LRD consisted of fiber ( ≤ 20 g), lactose ( ≤ 5 g), and fat ( ≤ 20% of total energy) restriction. Patients were analyzed in 5 visits after informed consent: V1, 2 weeks before CRT; V2, at CRT initiation; V3, at the third CT cycle; V4, after BT; V5, 3 months after treatment completion. LACC, locally advanced cervical cancer; CRT, chemoradiotherapy; AID, anti-inflammatory diet. LRD, low-residue diet; CT, chemotherapy; BT, brachytherapy. Created in BioRender. Castro-Eguiluz, D. (2026) https://BioRender.com/h12s358.

The study design is illustrated in Figure 1. Energy and protein requirements for all patients were based on the Mexican Nutritional Consensus and were individualized according to comorbid conditions (13). A nutritionist planned the individualized diets for patients in both groups. For the patients in the AID group, 30% of total energy was from fat, and patients were encouraged to increase intake of foods rich in anti-inflammatory compounds, soluble fiber, and probiotics. Only a selection of anti-inflammatory foods were provided to support adherence to the AID: green tea, chia seeds, ginger, and turmeric. For the LRD group, the nutritionist designed a diet low in fiber ( ≤ 20 g), lactose ( ≤ 5 g), and fat ( ≤ 20% of total energy intake). Both groups received counseling from a nutritionist on strategies for implementing the dietary interventions. Each patient received visual materials, including a dietary guide with the number of servings from each food group needed to meet energy and macronutrient requirements; examples of typical foods in each group; and a sample menu developed by the nutritionist with the patient to confirm understanding of the intervention. For a more detailed description of the dietary interventions and counseling sessions, see the Supplementary Material 1. The trial included five visits: V1: 2 weeks before CRT; V2: On the day CRT began; V3: After three cycles of CT; V4: After BT; V5: 12 weeks after BT.

### 2.5 Dietary assessment and anthropometric measurements

Dietary intake for each patient was evaluated through a 24-hour dietary recall at each visit. We used the food models to estimate portion sizes and conducted a thorough interview to record the preparation methods and any additional ingredients, ensuring a detailed recall. The Mexican System of Equivalent Foods was used to quantify energy, protein, fat, and carbohydrates during each evaluation. The Food Processor software (version 10.8.0.0) was employed to measure both the nutritive and non-nutritive components of the diet.

Weight was measured using a digital flat scale (SECA model 810; SECA Corp., Hamburg, Germany), while height was measured with a portable stadiometer (SECA model 213; SECA Corp., Hamburg, Germany). Body Mass Index (BMI) was calculated using the formula: BMI = weight (kg)/height (m)^2^.

### 2.6 Undernutrition assessment and body composition analysis

The Patient-Generated Subjective Global Assessment (PG-SGA) was utilized to evaluate nutritional risk (14). A patient was considered malnourished if she met two or more of the following criteria: PG-SGA categories B or C, BMI under 18.5 kg/m^2^, significant weight loss, albumin below 3.5 g/dL, hemoglobin below 12 g/dL, and energy intake below 90% of requirement (15).

Body composition was analyzed using cross-sectional computed tomography images of the third lumbar (L3) vertebra obtained before treatment and at 3 months post-treatment. Two trained researchers analyzed the images using HOROS software (USA, v. 4.0.1). The skeletal muscle (SM) area was calculated using Hounsfield Units (HU) between -29 and +150, SM was then normalized to the patient’s squared height (cm^2^/m^2^) to derive the skeletal muscle index (SMI), moderate sarcopenia was identified when SMI was below 38.5 cm^2^/m^2^; subcutaneous adipose tissue (SAT) area was defined between -190 and -30 HU; visceral adipose tissue (VAT) area was defined between -150 and -50 HU; total adipose tissue (TAT) was calculated adding the SAT and VAT areas. The areas were normalized to the patient’s squared height (cm^2^/m^2^). Muscle strength was evaluated through handgrip strength in the dominant hand using a dynamometer (Smedley T-18, Takei Scientific Instruments Co., Ltd., Niigata, Japan).

### 2.7 Gastrointestinal toxicity, systemic and intestinal inflammation

Gastrointestinal toxicity was evaluated at each visit using the Common Terminology Criteria for Adverse Events (CTCAE V.5) (16).

To assess the systemic and intestinal inflammatory response, blood and fecal samples were collected during visits 2, 3, and 5. Serum cytokine levels, specifically TNF-α, IL-6, IL-1β, IL-4, IFN-γ, IL-22, and IL-17A, were measured using the ProcartaPlex Human magnetic kit from Thermo Scientific. Fecal calprotectin levels were evaluated with the human S100A8/S100A9 Heterodimer Quantikine ELISA Kit from R&D Systems. All procedures followed the manufacturer’s protocol (17).

A complete blood count was performed, which included measurements of albumin, hemoglobin, serum proteins, and total leukocyte count. Blood samples were collected after fasting. The Prognostic Nutritional Index (PNI) (18) and the Systemic Inflammation Index (SII) (19) were calculated. G-CSF use was identified in our database; 4 patients (4.8%) received it, most at the 3rd QT cycle, and it did not change the SII results.

### 2.8 Response to treatment, proctopathy and overall survival

Response to treatment was evaluated in all patients using a contrast-enhanced computed tomography scan 3 months after completing BT. Tumor response was assessed according to the Response Evaluation Criteria in Solid Tumors (RECIST v1.1), with responses categorized as complete response (CR), partial response (PR), progressive disease (PD), or stable disease (SD).

After the final visit, patients were monitored every 6 months for the development of proctopathy, which was diagnosed and confirmed through colonoscopy. The Vienna Classification was used to grade proctopathy based on histopathological findings. Dates of CC diagnosis and either death or the last follow-up were used to calculate Overall Survival (OS) over a six-year follow-up period.

Health-related quality of life (HRQoL) was evaluated using the cancer questionnaire EORTC QLQ-C30 v.3, along with the specific CC module EORTC QLQ-CX24, both in the Spanish version adapted for Mexico (20, 21).

### 2.9 Statistical analysis

A Kolmogorov-Smirnov test was conducted to evaluate the normality of the quantitative variables. To assess differences in medians across the five time-point evaluations, the Friedman test was utilized. The Mann-Whitney U test was employed to compare differences between the two groups at each evaluation.

Qualitative variables were expressed as frequencies and percentages. The Q-Cochran test was used to evaluate differences in qualitative variables across the five time-point evaluations, while the chi-squared test assessed differences between the two groups.

A proctopathy curve was constructed from the beginning date of CRT until the date of proctopathy diagnosis or the last follow-up visit. Additionally, an OS curve was developed from the date of CC diagnosis to the date of death or the last follow-up visit. The Kaplan-Meier method was used to estimate survival fractions, and the log-rank test was employed to compare differences between groups.

For time-to-event outcomes, multivariate analyses were performed using Cox proportional hazards regression models to estimate hazard ratios and identify independent predictors while accounting for censored observations. Variables considered clinically relevant or statistically significant in univariate analyses were included in the multivariable models.

For longitudinal and repeated-measures outcomes, mixed-effects models were used to evaluate changes over time and to account for within-subject correlations by including both fixed and random effects.

Missing values in the database were imputed using Multivariate Imputation by Chained Equations and the Predictive Mean Matching method, configured with 15 iterations to ensure Markov chain convergence. The missing data sensitivity analysis is described in Supplementary Material 2. For the 35 imputed datasets, the pooling strategy depended on the models’ assumptions. For parametric models, we explicitly applied Rubin’s rules. The pooled parameter estimates were calculated by averaging the individual estimates across the 35 datasets, and the total variance was computed by combining the within-imputation and between-imputation variances to account for missing data uncertainty. The non-parametric tests were performed separately on each of the 35 datasets, and the median *p*-value is reported as the final estimate of statistical significance.

To estimate the effect size between the intervention groups for categorical outcomes, the Relative Risk (RR) and its 95% Confidence Interval (CI) were calculated. For continuous non-parametric variables, the Hodges-Lehmann estimator was used to determine the 95% CI of the median differences.

Data analysis was conducted using PRISM version 10 (GraphPad Software, Inc., San Diego, CA, USA) and SPSS version 23 for Mac (IBM., Armonk, NY, 2021), with a significance level set at *p* < 0.05.

## 3 Results

**FIGURE 2:**
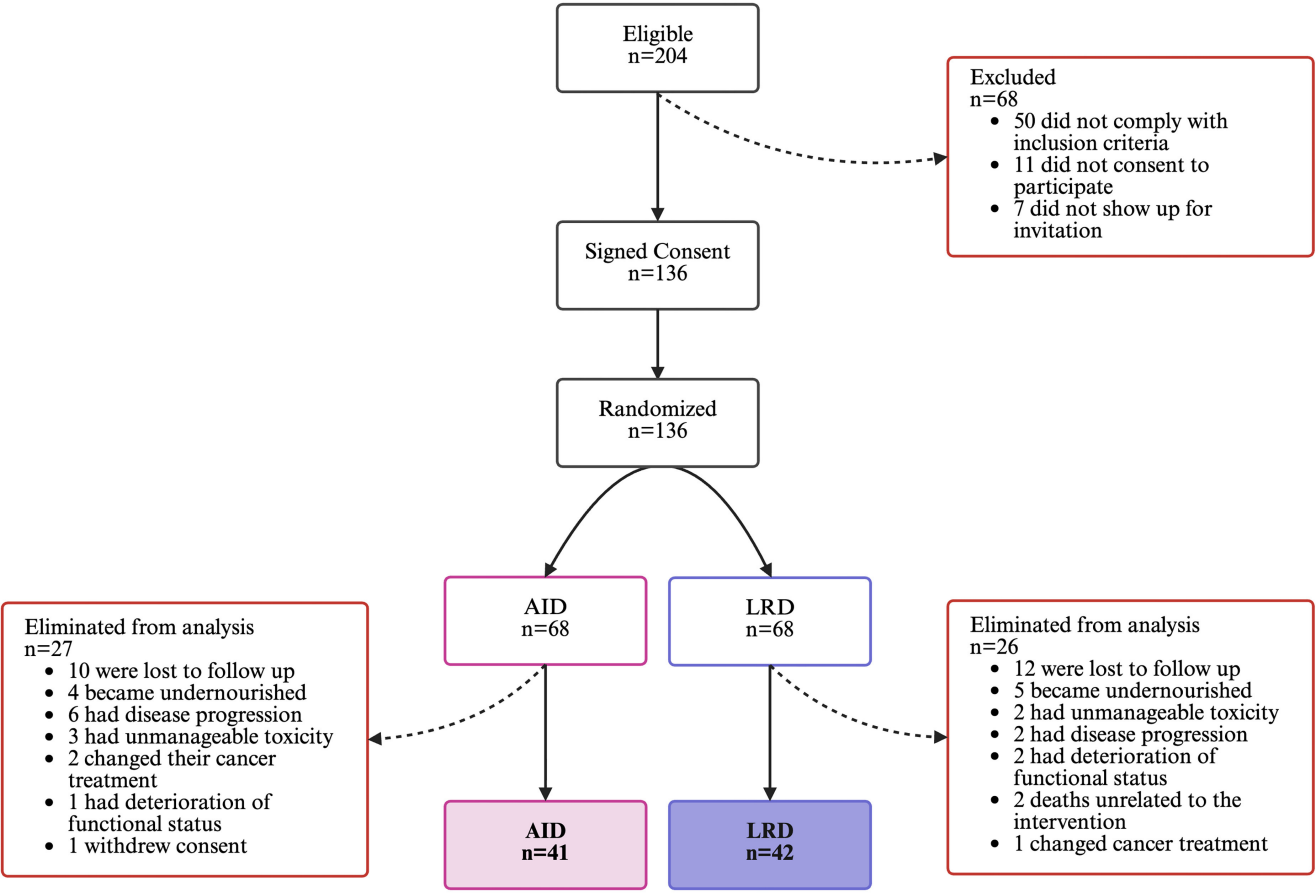
Flowchart of patients’ eligibility. AID, anti-inflammatory diet; LRD, low residue diet. Created in BioRender. Castro-Eguiluz, D. (2026) https://BioRender.com/nzwpqzg.

A total of 204 women diagnosed with LACC were initially screened for participation in the study (Figure 2). Of these, 68 did not meet the inclusion criteria. The primary reasons for exclusion were impaired renal function (22%), inability or unwillingness to follow the prescribed dietary regimen (16.1%), failure to attend the scheduled nutrition consultation (10.2%), and other reasons (51.7%). Out of 136 participants randomized into the AID or LRD, 83 were included in the final analysis, as 53 participants were either eliminated from the study or did not complete all required visits. A comparative analysis revealed that patients who were excluded from the study were more likely to be at an advanced stage (*p* = 0.002), had a significantly higher prevalence of renal impairment at diagnosis (26.4% vs. 6%; RR: 2.42; 95% CI: 1.137–5.154; *p* = 0.001), and were less likely to receive at least four cycles of CT (38.6% vs. 13.8%; RR: 0.424; 95% CI: 0.267–0.676; *p* = 0.001) compared to the included patients.

Table 1 describes the characteristics of the entire population and by dietary intervention group. No statistically significant differences were observed between the AID and LRD groups. After the initial assessment, patients were instructed to follow their assigned dietary intervention.

**TABLE 1:** Baseline characteristics of cervical cancer patients.

*Variables*	*All* *(n = 83)*	*AID* *(n = 41)*	*LRD* *(n = 42)*	*Difference (95% CI)^a^*	*p*
Age (years)^b^	45 (38–55)	45 (38.5–58)	46.5 (37.5–55)	−1 (−6,4)	0.964^c^
Geriatric patients, *n* (%)	12 (14.5)	7 (17.1)	5 (11.9)		0.503^d^
BMI (kg/m^2^)^b^	27.8 (25.2–32.1)	28.37 (25.4–31)	27.21 (24.6–32.6)	0.21 (−2.13, 2.43)	0.649^c^
ECOG, n (%)					
0	41 (49.4)	23 (56.1)	18 (42.9)		0.462^d^
1	38 (45.8)	16 (39)	22 (52.4)		
2	4 (4.8)	2 (4.9)	2 (4.8)		
Nutritional status by BMI, n (%)					
Low weight	1 (1.2)	1 (2.4)	0		0.326^d^
Normal	18 (21.7)	6 (14.6)	12 (28.6)		
Overweight	34 (41)	19 (46.3)	15 (35.7)		
Obese	30 (36.1)	15 (36.6)	15 (35.7)		
Stage of cervical cancer, n (%)					
IB3	8 (9.6)	3 (7.3)	5 (11.9)		0.792^d^
IIA	4 (4.8)	2 (4.9)	2 (4.8)		
IIB	19 (22.9)	11 (26.8)	8 (19)		
IIIB-IIIC	52 (62.7)	25 (61)	27 (64.3)		
Histology, n (%)					
Squamous	63 (75.9)	32 (78)	31 (73.8)		0.893^d^
Adenosquamous	18 (21.7)	8 (19.5)	10 (23.8)		
Other	2 (2.4)	1 (2.4)	1 (2.4)		
Chemotherapy, n (%)					
Cisplatin	77 (92.7)	37 (90.2)	40 (95.2)		0.576^d^
Gemcitabine	4 (4.8)	3 (7.3)	1 (2.4)		
Both	2 (2.4)	1 (2.4)	1 (2.4)		
Cycles of chemotherapy, n (%)					
<4	11 (13.3)	5 (12.2)	6 (14.3)		0.779^d^
≥4	72 (86.7)	36 (87.8)	36 (85.7)		
Radiotherapy, median (Q1–Q3)					
Radiotherapy dosage (Gy)^b^	50 (45–50.4)	50 (45–50.2)	50 (45–50.4)	0 (0,0)	0.672^c^
Brachytherapy dosage (Gy)^b^	28 (26–28)	28 (25.7–28)	26 (26–28)	0 (0,2)	0.600^c^
Comorbidities, n (%)					
Yes	46 (55.4)	23 (56.1)	23 (54.8)		0.903^d^
No	37 (44.6)	18 (43.9)	19 (45.2)		
Type 2 diabetes, n (%)					
Yes	8 (9.6)	3 (7.3)	5 (11.9)		0.713^d^
No	75 (90.4)	38 (92.7)	37 (88.1)		
Systemic arterial hypertension, n (%)					
Yes	11 (13.3)	5 (12.2)	6 (14.3)		0.436^d^
No	72 (86.7)	36 (87.8)	36 (85.7)		
Renal deterioration, n (%)					
Yes	5 (6)	3 (7.3)	2 (4.8)		0.625^d^
No	78 (94)	38 (92.7)	40 (95.2)		

AID, anti-inflammatory diet; LRD. low residue diet; BMI, body mass index; ECOG, Eastern Cooperative Oncology Group. ^a^Hodges-Lehmann estimator for median differences, ^b^Median (Q1-Q3), ^c^Mann-Whitney U test, ^d^Chi-square test or Fisher’s exact test.

### 3.1 Characteristics of nutrient consumption

**FIGURE 3:**
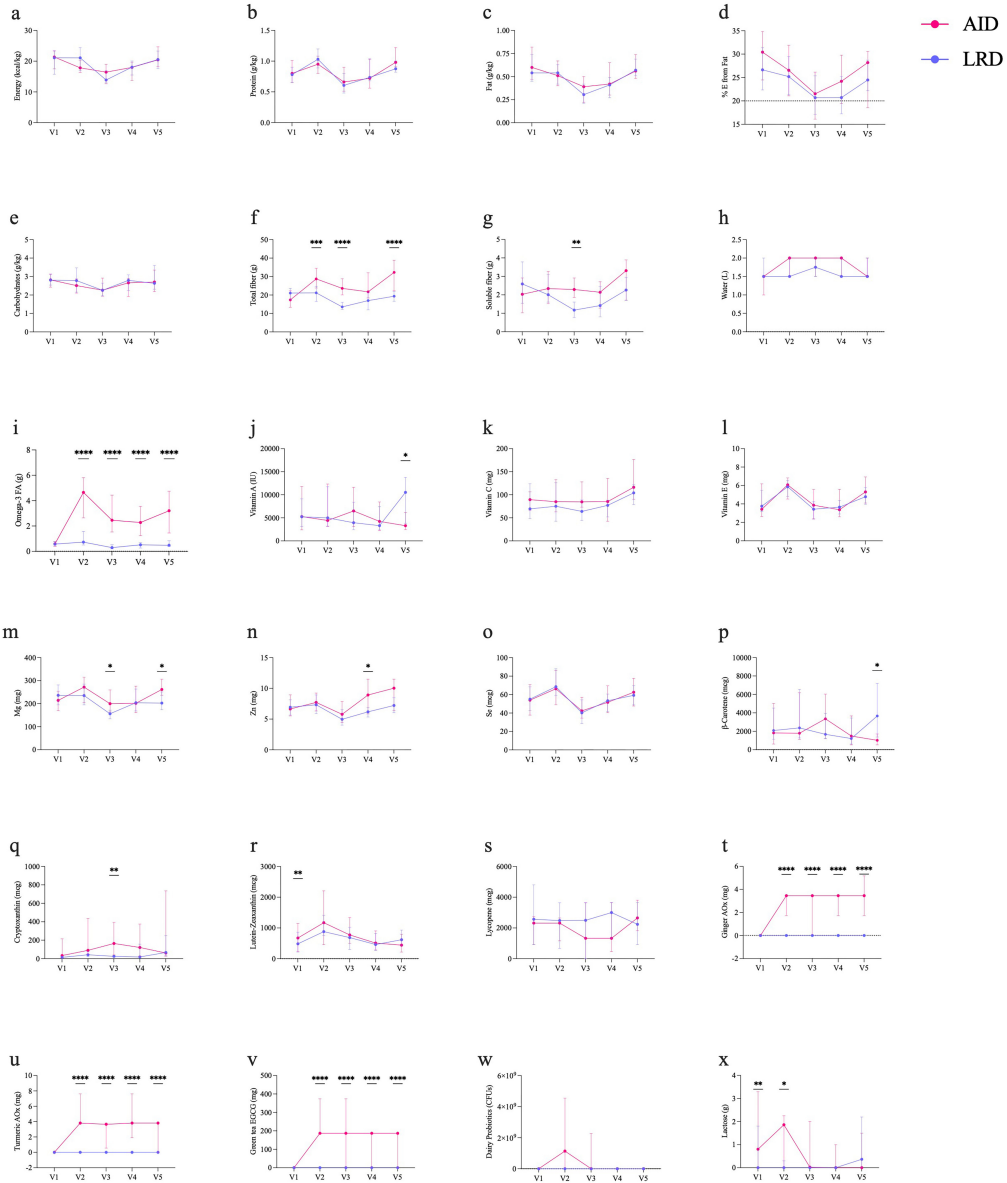
Dietary assessment during the intervention. **(A–X)** Nutrient intake quantified for participants in the AID (pink line) and the LRD (blue line) group through the five visits. Data are presented as median (95% CI). Mann-Whitney U Test was used to compare differences among groups. *p < 0.05; **p < 0.01; ***p < 0.001; ****p < 0.0001. AID, anti-inflammatory diet; LRD, low residue diet.

Dietary assessments were conducted before and during treatment to investigate previous dietary patterns and baseline nutrient consumption (Supplementary Table 1). The mean dietary adherence was 83.2% (SD, 14.9%) in the AID group and 89.4% (SD, 7.8%) in the LRD group. Figure 3 depicts the characteristics of the dietary interventions and the main differences observed during the five evaluations of the study. The consumption of energy, protein, fat, and carbohydrates was similar among groups across the five visits (Figures 3a–e). Both groups demonstrated significant temporal changes, particularly a reduction in energy and protein intake at V3. While fat (*p* = 0.362) and carbohydrate (*p* = 0.065) intake remained stable in the AID group, significant reductions were observed in the LRD group (*p* = 0.004 for fat and *p* = 0.028 for carbohydrates). Water consumption tended to be higher in the AID group (Figure 3h). The AID group consistently consumed more dietary fiber throughout the treatment (Figure 3f). The intake of soluble fiber was notably higher in the AID group compared to the LRD group from visits 2 to 5 (Figure 3g).

We also assessed the intake of anti-inflammatory nutritive, and non-nutritive compounds in each group. The AID group consumed significantly more omega-3 FA than the LRD group (Figure 3i). Regarding antioxidant vitamins A, C, and E, intake was comparable between the groups (Figures 3j–l). At visit 5, vitamin A intake was higher in the LRD group compared to the AID group (Figure 3j). Differences were also noted in the consumption of immune-modulatory and antioxidant minerals. The AID group had a higher intake of magnesium and zinc, but not selenium, compared to the LRD group (Figures 3m–o). Interestingly, β-carotene intake was lower in the AID group at visit 5 (Figure 3p).

Furthermore, the AID group showed greater consumption of cryptoxanthin and lutein–zeaxanthin (Figures 3q–r). Non-nutritive anti-inflammatory compounds, including antioxidants from ginger, turmeric, and green tea, were consumed in significantly larger amounts by the AID group compared to the LRD group (Figures 3t–v).

Although dairy probiotics were recommended in the AID, patients only consumed them by visit 2, and neither group consumed dairy products during the rest of the intervention period (Figures 3w, x).

While the consumption of most nutrients varied significantly across visits, we observed a time-by-dietary arm interaction effect on the intake of fiber, soluble fiber, omega-3 FA, and antioxidants from ginger, turmeric, and green tea (Supplementary Table 2). Overall, these results indicate that, despite similar energy and macronutrient intake, the AID group consumed significantly higher amounts of various anti-inflammatory dietary compounds than the LRD group.

### 3.2 Nutritional status and body composition

**FIGURE 4:**
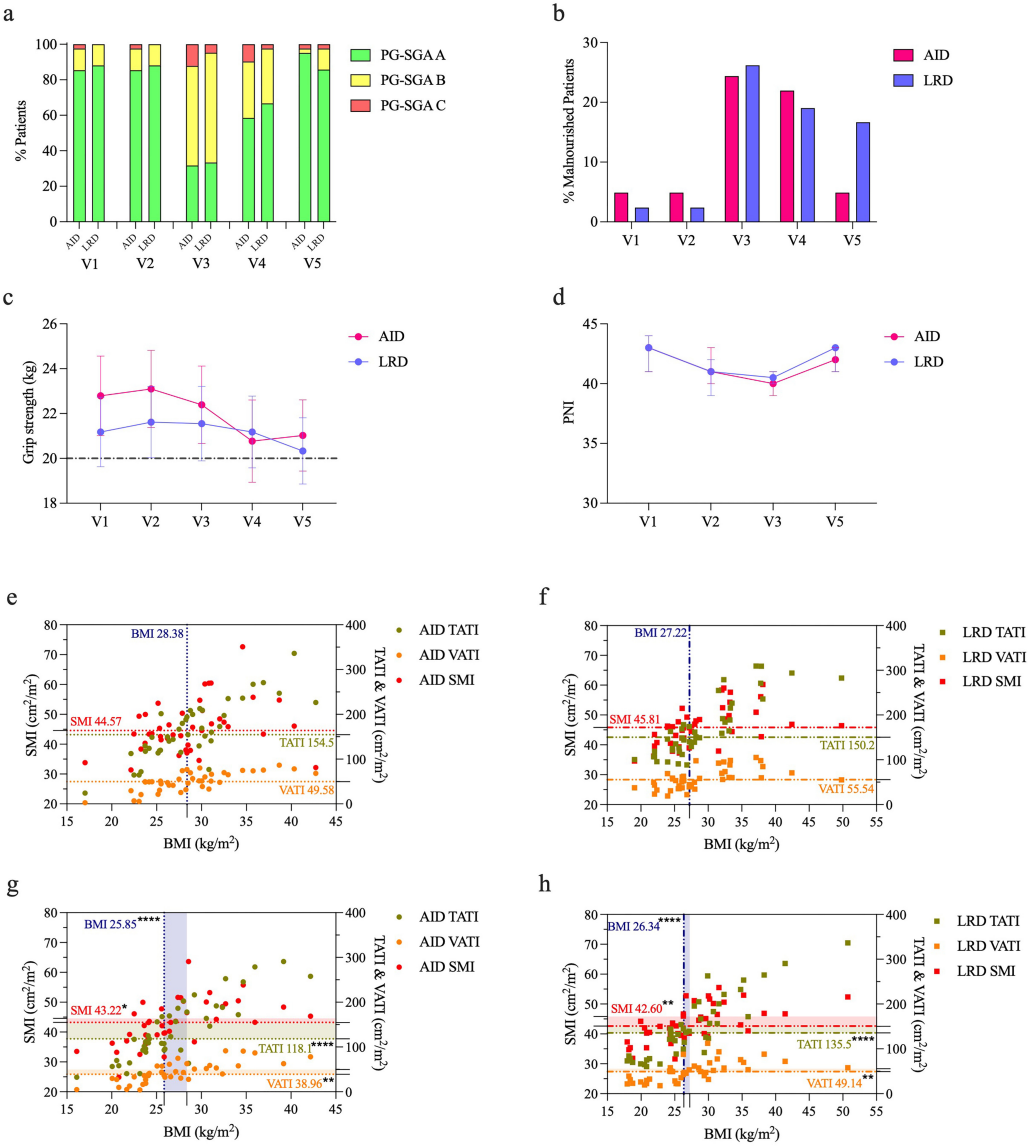
Nutritional assessment during the intervention. **(a)** %Patients that scored PG-SGA A (green bar), PG-SGA B (yellow bar), and PG-SGA C (orange bar) in participants from the AID and the LRD groups through the five visits. **(b)** %Malnourished patients from the AID and the LRD groups through the five visits. **(c)** Median (95% CI) handgrip strength in participants from the AID and the LRD groups through the five visits (gray dotted line indicates the cutoff value for women). **(d)** Median (95% CI) PNI in participants from the AID and the LRD groups through visits 1, 2, 3, and 5. **(e–h)** SMI (red dots), TATI (green dots) and VATI (orange dots) in relation to BMI for each participant in the AID and LRD groups. Dotted lines indicate the median values for BMI (blue); SMI (red); TATI (green); and VATI (orange). **(e)** AID group at Visit 1. **(f)** LRD group at Visit 1. **(g)** AID group at Visit 5. **(h)** LRD group at Visit 5. AID, anti-inflammatory diet; LRD, low residue diet; PG-SGA, patient generated-subjective global assessment; PNI, prognostic nutritional index; BMI, body mass index; SMI, skeletal muscle index; TATI, total Adipose tissue index; VATI, visceral adipose tissue index. *p < 0.05; **p < 0.01; ****p < 0.0001 comparing visit 5 to visit 1 **(e-h)**.

Nutritional status is partly determined by dietary intake; therefore, we evaluated whether the AID and LRD interventions had a nutritional impact throughout the study period. The findings related to nutritional status are summarized in Figure 4.

The PG-SGA was used to evaluate the overall nutritional condition (Figure 4a). The risks of undernutrition, as assessed by scores B or C, were not statistically significant at any visit (Supplementary Table 3). For both groups, the worst scores were observed at visit 3 (AID: 68% vs. LRD: 66%), with significant improvements noted by visit 5 (AID: 5% vs. LRD: 14%). These changes over time were statistically significant in both groups (*p* < 0.0001). While the LRD group had a higher proportion of patients classified as undernourished (score C), no statistically significant differences were found between the groups at any visit; at visit 5, the AID group showed a trend toward decreased risk (RR: 0.3415; 95% CI: 0.0815 to 1.383; *p* = 0.2646) (Figure 4a).

Undernutrition was assessed using anthropometric, biochemical, and dietary measures. The highest prevalence of undernutrition was recorded at visit 3 (Figure 4b); no increased risk was identified for either dietary group (AID RR: 0.93; 95% CI: 0.44 to 1.95; *p* > 0.9999). Nonetheless, the trend toward nutritional recovery was more pronounced in the AID group (Figure 4b). Improvement was observed by visit 5, where the risk of undernutrition was lower in the AID group compared to the LRD group, although this between-group difference did not reach statistical significance (RR: 0.29; 95% CI: 0.06 to 1.33; *p* = 0.155). Changes over time were statistically significant in both groups (AID: *p* = 0.002; LRD: *p* < 0.001).

Muscle strength, an indirect indicator of nutritional balance and metabolic stability, was assessed using handgrip strength (Figure 4c). The AID group consistently demonstrated higher strength than the LRD group but experienced a more abrupt decline by visit 4, followed by recovery by visit 5 (Figure 4c). The LRD group maintained strength throughout treatment, with a slight decline by visits 4 and 5. Changes over time were statistically significant in both groups (AID: *p* < 0.0001; LRD: *p* = 0.014), and we observed a significant time-by-dietary arm interaction effect (Supplementary Table 2).

The PNI, which is based on biochemical parameters and reflects nutritional adequacy, also decreased by visit 3 in line with the PG-SGA results, but showed recovery by visit 5 in both groups. Significant changes were observed over time in both groups (*p* < 0.0001) (Figure 4d).

Changes in body composition, SMI, TATI and VATI, in relation to BMI were analyzed (Figures 4e–h). At visit 1, in both groups, a significant proportion of patients were overweight or obese but maintained adequate SMI, with no significant cases of sarcopenic obesity detected (Figures 4e, f). By visit 5, changes in BMI were more pronounced in the AID group, associated with an important decrease in TATI and VATI, but only a slight decrease in SMI (Figure 4g). On the other hand, by visit 5, the LRD group had a smaller decrease in BMI, but it was importantly attributed to a higher SMI loss (Figure 4h).

Given that not all of the initial 136 patients were evaluated per ITT in the post-elimination visits, we used a variable available to most randomized patients: the MMI, obtained from the CT scan at 3 months after treatment completion for all patients (Supplementary Table 4); no differences were observed in MMI between groups. Next, a general linear model with repeated measures was used to evaluate changes in MMI from visit 1 to visit 5, with the diet group as a between-subject factor and relevant clinical covariates included. This analysis included 48 patients in the AID group and 55 in the LRD group because only participants with both initial and final CT scans were included. There was no significant interaction between time and diet group (*p* = 0.688), indicating that MMI evolution was similar across groups during the follow-up period. In the adjusted model, treatment response (*p* = 0.016) and the use of a radiosensitizer (*p* = 0.045) were significantly associated with changes in MMI, whereas the other covariates (number of CT cycles, RT dose, BT dose, presence of comorbidities, renal deterioration, and tumor stage) did not show a significant association. These results are consistent with the longitudinal per-protocol analysis and allow us to conclude that the diet group does not significantly modify the evolution of MMI over time, even after adjusting for covariates.

To further prove this point, we performed analyses per protocol and per ITT. The first is a multivariable model of variables independently associated with malnutrition (Supplementary Table 5). This model shows that the diet group was not independently associated with the development of malnutrition. The next model is per ITT (Supplementary Table 6). This model also shows that the diet group was not independently associated with malnutrition, but only the presence of comorbidities.

### 3.3 Gastrointestinal toxicity

**FIGURE 5:**
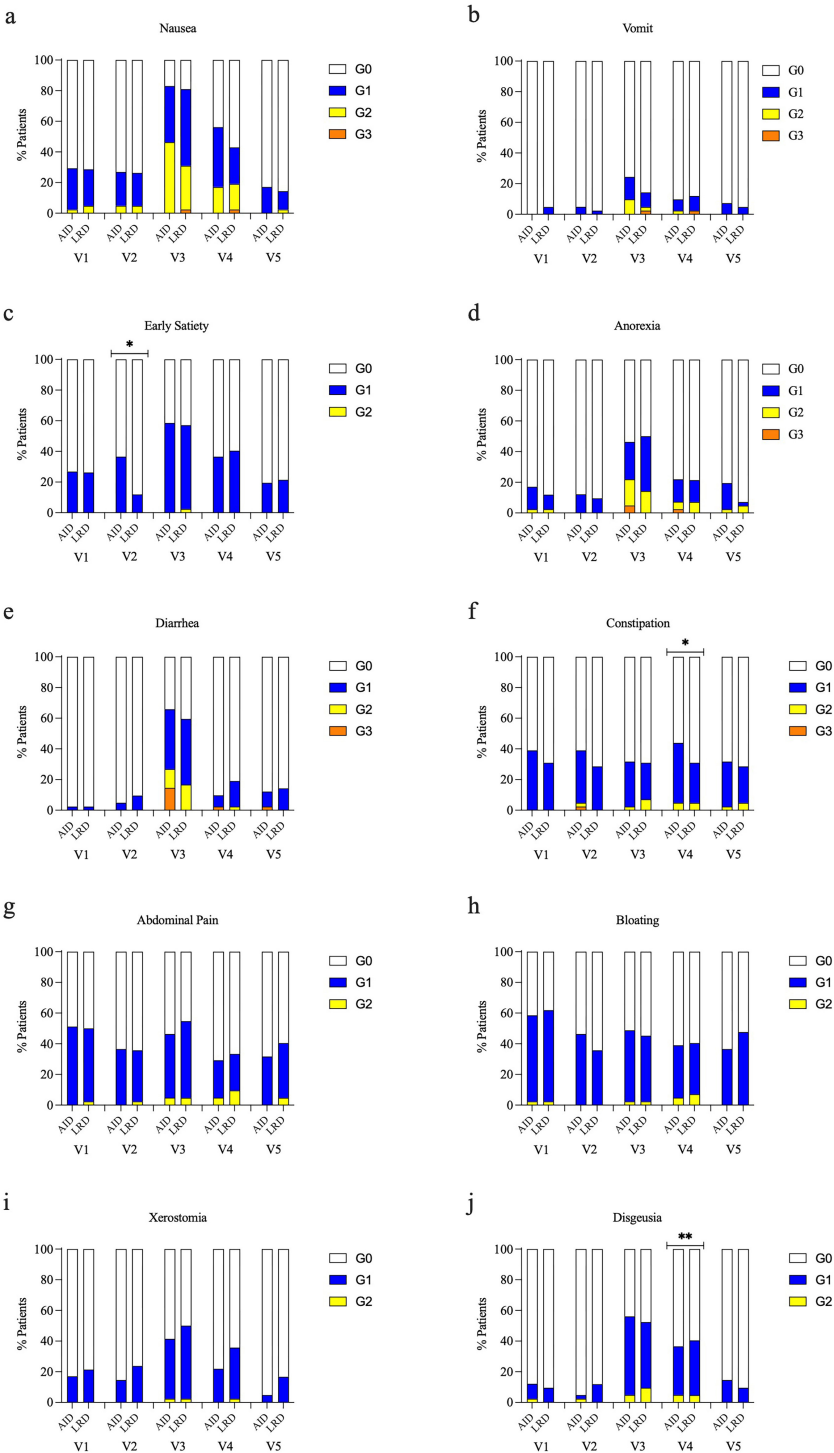
Toxicity assessment during the intervention. **(a–j)** %Patients from the AID and the LRD group who reported gastrointestinal symptoms severity through the five visits. Chi-squared test or Fisher’s exact test was used to compare proportions among groups. *p < 0.05; **p < 0.01. AID, anti-inflammatory diet; LRD, low residue diet; G0, Grade 0 (no symptom, white bar). G1 (blue bar), G2 (yellow bar), and G3 (orange bar): symptoms severity graded according to the CTCAE V.5 (Common Toxicity Criteria for Adverse Events).

As previously mentioned, undernutrition is a multifactorial condition. In our patient population, the main causes involve tumor metabolism and toxicity resulting from oncological treatment. Figure 5 presents the main gastrointestinal toxicities reported by patients during the five study visits, categorized by severity grade. The effect sizes were calculated for the development of toxicity grades ≥ 2, and no statistically significant effects were observed (Supplementary Table 7).

At baseline, prior to the dietary interventions, gastrointestinal symptoms were generally mild and similar across both groups. The most frequently reported symptoms were likely related to the tumor and included constipation, abdominal pain, and bloating (Figures 5f–h). By visit 2, a significant increase in grade 1 early satiety was reported in the AID group (Figure 5c), likely due to the substantial increase in fiber intake after the AID was implemented.

Visit 3 exhibited the highest frequency of toxicities, particularly nausea (AID: 83% vs. LRD: 81%); however, no significant differences between dietary groups were observed. Regarding the severity of toxicity, most patients had primarily grade 1 and 2 symptoms. Notably, during this visit, some patients in the AID group reported grade ≥ 2 anorexia (RR: 1.537; 95% CI: 0.6235–3.847; *p* = 0.4052) and diarrhea (RR: 1.610; 95% CI: 0.7132–3.703; *p* = 0.2969) (Figures 5d, e), while those in the LRD group reported grade ≥ 2 nausea and vomiting (Figures 5a, b).

By visit 4, most patients experienced a reduction in symptoms. However, the AID group showed a slight increase in mild constipation (44%), significantly higher than that reported by the LRD group (31%); however, no significant effect was observed in grade ≥ 2 constipation (RR: 1.024; 95% CI: 0.1869–5.610; *p* > 0.9999) (Figure 5f). Changes over time did not differ significantly in either intervention group. Conversely, the LRD group reported significantly more cases of dysgeusia than the AID group (Figure 5j), with significant variation observed over time in both groups (Supplementary Table 2).

By visit 5, most patients experienced no symptoms, and any present were mostly mild. Nevertheless, some patients in the LRD group continued to report grade 2 nausea, anorexia, constipation, and abdominal pain (Figures 5a, d, f, g). It is noteworthy that patients in the AID group consistently exhibited a trend of lower abdominal pain compared to the LRD group. The reduction in pain observed in the AID group was statistically significant over time (*p* = 0.034). All symptoms, except constipation, varied significantly over time; however, no time-by-dietary arm interactions were observed (Supplementary Table 2).

### 3.4 Markers of systemic and local inflammation

**FIGURE 6:**
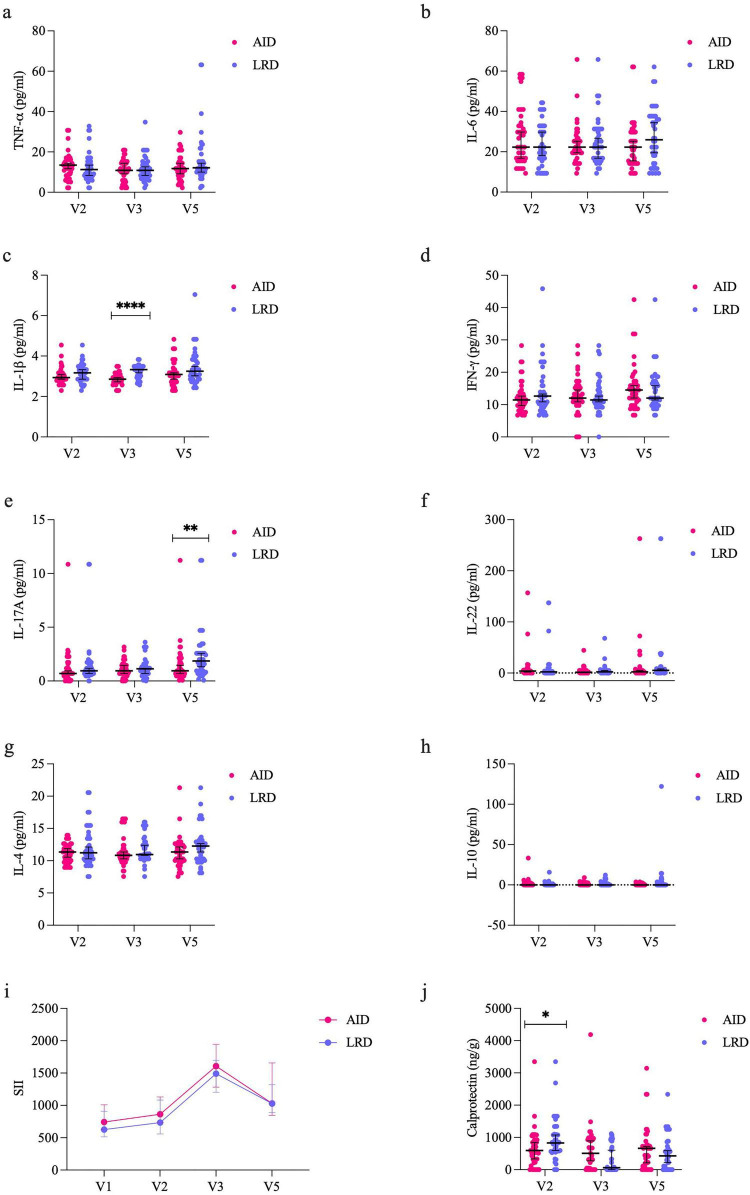
Assessment of systemic and intestinal inflammation during the intervention. **(a–h)** Scatter plot of serum cytokine levels in participants from the AID and the LRD groups, at visits 2, 3, and 5 (bar indicates median value ± 95% CI). **(i)** Median (95% CI) SII in participants from the AID and the LRD groups, at visits 1, 2, 3, and 5. **(j)** Scatter plot of fecal calprotectin levels in participants from the AID and the LRD groups, at visits 2, 3, and 5 (bar indicates median value ± 95% CI). Mann-Whitney *U*-test was used to compare among groups. *p < 0.05; **p < 0.01; ****p < 0.0001. AID, anti-inflammatory diet; LRD, low residue diet; TNF, Tumor necrosis factor; IL, Interleukin; IFN, Interferon; SII, systemic inflammatory index.

We evaluated several inflammatory parameters to assess the immune-modulating effects of the dietary interventions and their potential influence on gastrointestinal toxicity (Supplementary Table 8). Figure 6 illustrates the variations in serum cytokines and fecal calprotectin levels at visits 2, 3, and 5.

At visit 2, before treatment, no significant differences were observed in cytokine levels between the intervention groups. However, the LRD group displayed higher levels of inflammatory cytokines, specifically IL-1β (Figure 6c) at visit 3 and IL-17A (Figure 6e) at visit 5. These cytokines are crucial in chronic systemic inflammation and the mucosal inflammatory response. The LRD group demonstrated a progressive increase in IL-17A levels over time, with significant differences between visits 2 and 3 (*p* = 0.007) and between visits 3 and 5 (*p* = 0.006). Conversely, the AID group exhibited a significant increase in IL-1β levels from visit 3 to visit 5 (*p* = 0.006).

Levels of other cytokines, including TNF-α, IL-6, IFN-γ, IL-22, IL-4, and IL-10, remained consistent throughout the intervention period (Figure 6). We observed that IL-1β and IFN-γ varied significantly over time and across dietary arms; however, no time-by-dietary arm interaction was observed (Supplementary Table 2).

We also analyzed the SII (Figure 6i), which incorporates neutrophil, lymphocyte, and platelet counts. SII levels increased for all patients at visit 3 during oncological treatment and decreased by visit 5. While there were no significant differences among the groups, significant differences over time were noted within both groups (*p* < 0.0001). This increase in immune cell count reflects the extent of the inflammatory response associated with CRT.

To assess local intestinal inflammation, we measured fecal calprotectin levels (Figure 6j). Before treatment, at visit 2, the LRD group had significantly higher levels of calprotectin. These levels were reduced at visit 3 but increased again by visit 5 after treatment (*p* = 0.009). In contrast, the AID group maintained consistent calprotectin levels throughout treatment. Here, we also observed a significant time-by-dietary arm interaction (Supplementary Table 2).

### 3.5 Response to treatment

Considering that nutritional status, body composition, and related nutritional parameters can influence response to treatment, we evaluated whether dietary interventions affected this outcome. In the AID group, 29 patients (70.7%) achieved CR, while 12 patients (29.3%) had either a PR, SD, or PD. In the LRD group, 23 patients (54.8%) achieved CR, whereas 19 patients (45.2%) had PR, SD, or PD. However, the differences in tumor response between the two dietary groups were not statistically significant (RR: 1.29; 95% CI: 0.92–1.8; *p* = 0.133).

### 3.6 Proctopathy and overall survival

**FIGURE 7:**
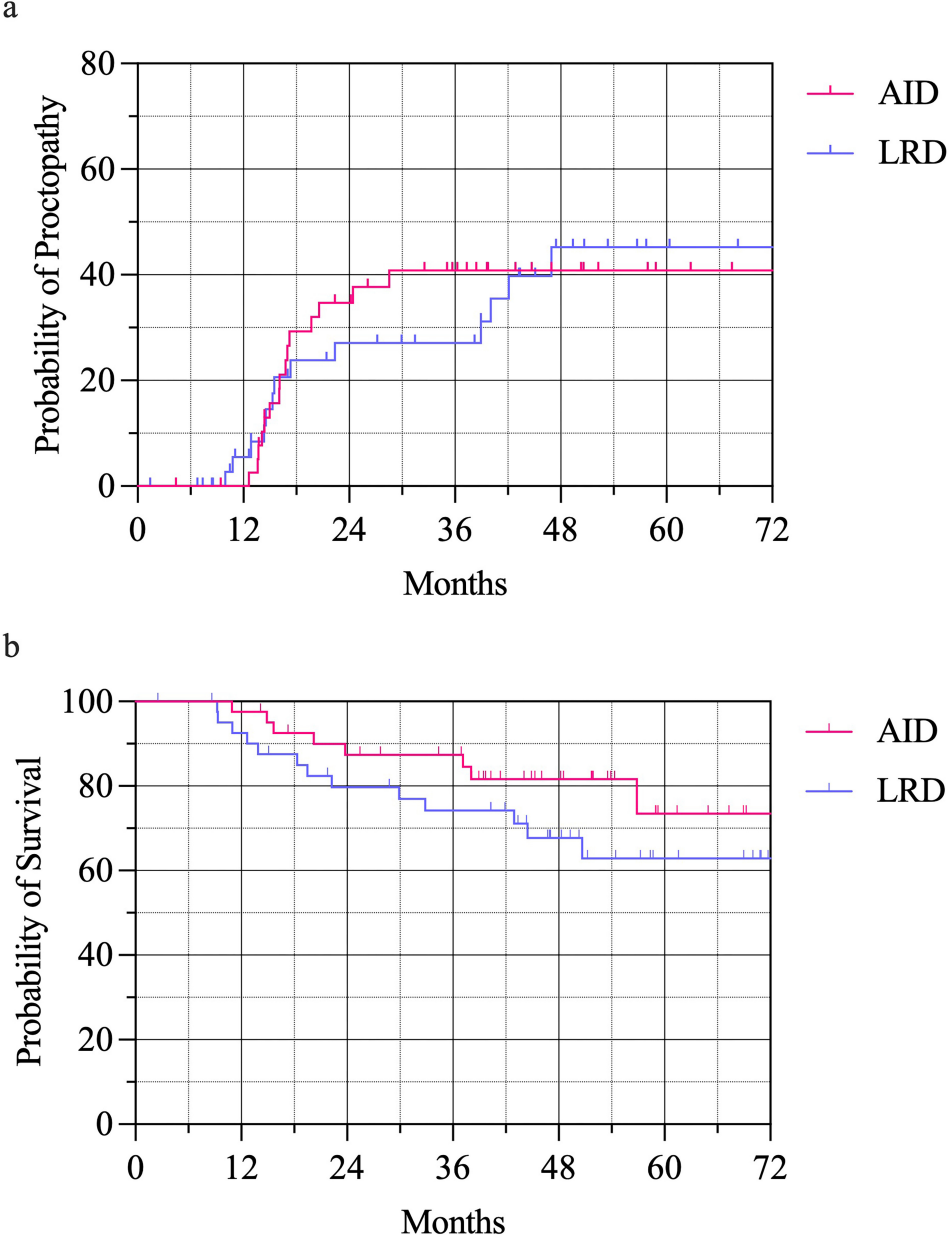
Development of proctopathy and overall survival in participants at 6-year follow-up. **(a)** Probability of proctopathy in participants from the AID and the LRD groups. **(b)** Probability of survival in participants from the AID and the LRD groups. Kaplan-Meier method was used to estimate survival. Log-Rank test was used to compare differences among groups. AID, anti-inflammatory diet; LRD, low residue diet.

Chronic intestinal inflammation can lead to tissue damage, functional impairment, and health complications. Proctopathy is a late-onset toxicity that may be diagnosed months after pelvic RT. We evaluated the incidence of this condition in both groups (Figure 7a). The median follow-up period for the entire population was 27.1 months, during which 28 patients (33.7%) developed proctopathy, 14 patients (34.1%) in the AID group, and 14 patients (33.3%) in the LRD group. No significant differences were observed in the incidence (RR: 1.02; 95% CI: 0.56–1.87; *p* = 0.938), severity (RR: 1.537; 95% CI: 0.701–3.367; *p* = 0.536), or time to diagnosis (HR: 1.092; 95% CI: 0.526–2.266; *p* = 0.734).

OS was also assessed (Figure 7b). The median follow-up period for this assessment was 44.4 months. During this time, 8 patients (19.5%) in the AID group and 13 patients (31%) in the LRD group died. A trend indicated that the AID group experienced events later than the LRD group, but no significant differences were observed during this follow-up period (*p* = 0.152). A Cox regression model was used to assess variables independently associated with OS. In the bivariate analysis, the LRD was independently associated with higher mortality risk (HR: 3.181; 95% CI: 1.025–9.869; *p* = 0.045); however, this association was not maintained in the multivariate model. Response to treatment was independently associated with mortality risk in the multivariate model; disease progression had the highest mortality risk (HR: 18.773; 95% CI: 4.914–71.717; *p* < 0.0001). Other variables, such as clinical stage, comorbidities, and age, were not independently associated with OS.

### 3.7 The health-related quality of life improved across all groups

**FIGURE 8:**
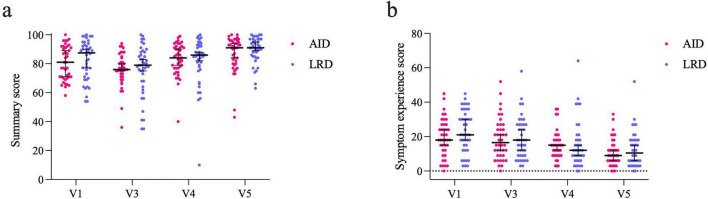
Health-related Quality of Life. **(a)** Scatter plot of summary score of participants in the AID and LRD groups, at visits 1, 3, 4, and 5 (bar indicates median value ± 95% CI). **(b)** Scatter plot of symptom experience score of participants in the AID and LRD groups, at visits 1, 3, 4, and 5 (bar indicates median value ± 95% CI). AID, anti-inflammatory diet; LRD, low residue diet.

Factors such as tumor-related symptoms, treatment-related toxicity, nutritional status, dietary intervention, and other patient-specific variables may impact HRQoL. Based on the summary score from the QLQ-C30 (Supplementary Table 9), a similar pattern across visits was observed in both dietary groups (Figure 8). QoL began with comparable scores (85 vs. 81, *p* = 0.688), declined during oncological treatment (78 vs. 77, *p* = 0.809), and gradually improved at visits 4 (86 vs. 80, *p* = 0.045) and 5 (93 vs. 91, *p* = 0.097) (Figure 8a). These changes were significant over time for both groups (*p* < 0.0001); however, no significant time-by-dietary arm interaction effects were observed (Supplementary Table 2). The EORTC QLQ-Cx24 was used to evaluate the QoL specifically of LACC patients (Supplementary Table 9). Although there were no significant differences between the groups, improvements were noted over time in sexual activity and sexual enjoyment (Supplementary Table 9), and symptom experience (Figure 8b) in both groups (*p* < 0.05).

## 4 Discussion

Evidence highlights the protective effects of nutrition during cancer treatment. Poor nutrition has been linked to increased treatment toxicity, side effects that limit treatment dosage, reduced therapeutic responses, and lower OS rates in patients undergoing surgery, radiotherapy, and systemic therapies, including chemotherapy, targeted treatments, and immunotherapy, as well as during the end-of-life stage (22). Nutritional status encompasses not only dietary intake but also body composition, including skeletal muscle mass and adipose tissue, as well as the availability of vitamins, minerals, and trace elements. These components fulfill vital structural and metabolic functions and are involved in tissue repair mechanisms, and the absorption, distribution, metabolism, and elimination of cancer treatments (23–25).

While the human diet is primarily composed of macronutrients, the role of food-derived chemicals, such as antioxidants, cannot be overlooked, particularly in cancer prevention. It is estimated that over 7,000 bioactive compounds are found in foods, although most remain uncharacterized, and their biological effects on humans are still largely unknown (26). Some compounds act synergistically and can influence physiological processes, such as gut microbiota modulation and inflammation regulation. For instance, the Western dietary pattern, which is high in ultra processed foods, additives, and other pro-inflammatory ingredients, contrasts with the Mediterranean dietary pattern. The Mediterranean diet, rich in legumes, bread, fish, and nuts, is associated with a lower prevalence of opportunistic bacteria, reduced endotoxemia, and decreased inflammation.

Similarly, the anti-inflammatory dietary pattern that includes nuts, oily fish, fruits, vegetables, and whole cereals has been linked to increased production of SCFAs. These metabolites are known to support gut barrier integrity and exert anti-inflammatory effects (27). Such foods are recognized for their high content of polyphenols, vitamins, minerals, monounsaturated fatty acids (MUFA), and fiber, which serves as a prebiotic, promoting SCFA production in the gut, and polyunsaturated fatty acids (PUFAs), including omega-3 FA. These compounds can modulate gene expression, have antioxidant properties, influence immune cell activity, and foster an anti-inflammatory environment in most tissues (28). In this study, the AID group had a higher intake of fiber, omega-3 FA, and antioxidants, confirming its anti-inflammatory characteristics. Since the AID includes anti-inflammatory foods that are not typical of Mexican cuisine and may be unfamiliar to patients, we introduced these foods at their first visit to ensure understanding and compliance. Although providing these specific foods could be seen as influencing their choices, it encouraged them to obtain more of these beneficial foods themselves when needed, demonstrating the importance and effectiveness of this approach.

In contrast, the LRD group followed a restrictive diet low in fiber, lactose, and fats. The LRD involves eliminating certain foods, and patients are more familiar with this approach because it has traditionally been recommended for several gastrointestinal issues, so there is no need to provide different foods. Some studies suggest that diets low in fermentable carbohydrates may alleviate gastrointestinal symptoms and improve quality of life. However, these outcomes could also stem from broader dietary changes, improved food hygiene, and a reduced consumption of ultra processed foods. Nonetheless, such restrictive diets are not without drawbacks. Prolonged adherence to these diets may lead to nutritional deficiencies, a decline in nutritional status, dysbiosis, and recurrence of symptoms (29). While the fats in the LRD mainly come from PUFAs, our study found a higher intake of omega-6 FA and saturated fats, along with a tendency for increased refined sugar consumption, factors associated with dysbiosis and a subsequent pro-inflammatory response (30). Participants in our study struggled to maintain fat intake below 20% of total energy, even at Visit 3, when symptoms were at their worst. Notably, energy and protein intake were similarly affected in both groups throughout the study. In fact, protein intake significantly deteriorated by Visit 3 in both groups, with requirements being met at approximately 50% of the recommendation.

Interestingly, the LRD group had a significantly higher intake of Vitamin A at Visit 5. This may be explained by the liberalization of their restrictive diet 3 months post-treatment, during which patients frequently reintroduced local, vitamin A-rich foods (such as orange-colored vegetables or eggs) that had been avoided due to fat or fiber restrictions.

Low energy and protein intake led to a deterioration of nutritional status in both groups. The highest prevalence of undernutrition was observed at Visit 3, which was associated with a loss of SMI and a reduction in BMI. It is noteworthy that handgrip strength declined in both groups; however, it improved by Visit 5 in the AID group. In our study, the prevalence of both undernutrition and sarcopenia was lower compared to a previous report involving a similar cohort of patients who received only elemental restrictive nutritional recommendations without a structured individualized dietary intervention. These earlier recommendations consisted of a list of allowed foods (high-sugar, very low-fiber, and low-fat) and prohibited foods (high-fiber and high-fat). In that study, the prevalence of undernutrition reached 69% after BT, with sarcopenia observed in 58% of patients 3 months post-BT (3). To determine whether either of the dietary interventions investigated in this study was better than no dietary intervention, we performed a multivariate analysis comparing the data from the study mentioned (3) with our results. We found that AID and LRD (even among those with low adherence to the intervention) protected against undernutrition. Our study did not reveal statistically significant differences in the incidence of undernutrition between the dietary groups, meaning that neither diet provided superior protection against undernutrition. However, by Visit 5, there was a trend indicating a lower prevalence of undernutrition in the AID group compared to the LRD group. Low adherence to the diets seems to be linked to the presence of gastrointestinal toxicity, as only one patient in the cohort reported no gastrointestinal symptoms. The other participants experienced multiple symptoms and a significantly reduced energy intake, with some consuming as low as 3.5 kcal/kg, making weight maintenance unfeasible.

It is unsurprising that patients experience symptoms that limit their food intake due to CRT administration. The mechanism of action of CRT is associated with an increase in pro-inflammatory cytokines resulting from the death of neoplastic cells. This process triggers immune-cell activation of transcription factors, such as NF-kB, and leads to an elevated production of reactive oxygen species, mechanisms linked to metabolic alterations affecting various tissues and organs, including the central nervous system. Anorexia has been described as a consequence of neural inflammation and is directly involved in the deterioration of food intake. Furthermore, the increased catabolic state induces muscle breakdown and sarcopenia (1, 31). While we did not expect that diet alone could fully mitigate the adverse effects of such aggressive systemic treatment as CRT, the results of this study suggest that it is indeed possible to alleviate some symptoms.

The most common symptoms observed in this study were nausea, anorexia, and diarrhea, these primarily hinder food intake. In research related to pelvic RT, diarrhea has received particular attention due to its potential to lead to life-threatening complications rapidly. In our study, we found the same frequency of diarrhea across both interventions, suggesting that, contrary to popular belief, fiber does not lead to increased symptom development. However, there was a trend toward greater severity of diarrhea among patients in the AID group. Therefore, there is no need to reduce fiber intake during CRT unless severe diarrhea occurs; in such cases, a temporary reduction in fiber may help alleviate the symptom.

A similar conclusion was reached in another study (32), where no differences in intestinal symptoms were noted when comparing low, standard, and high-fiber diets. Furthermore, 1 year after dietary interventions, toxicity was lower in patients who followed a high-fiber diet compared to those in other groups. Both studies indicate that while it is impossible to prevent the onset of symptoms completely, patients can recover within a short time period with appropriate nutritional interventions, as we observed 3 months following treatment.

Patients in our study frequently reported additional symptoms such as abdominal pain, bloating, and constipation; however, these symptoms did not improve with dietary modifications. They appear to be clinical manifestations reflecting pathological changes in the intestinal mucosa (33).

To evaluate the anti-inflammatory potential of the dietary interventions, we measured local inflammation via fecal calprotectin levels and assessed systemic inflammation through serum cytokine analysis. Before treatment, the LRD group exhibited higher levels of fecal calprotectin. However, these values significantly decreased during treatment, only to rise again 3 months after treatment completion. To explore possible explanations for these findings, we conducted several correlation analyses. We noted that total energy intake at Visit 3 was positively correlated with fecal calprotectin levels at the same time point (*r* = 0.426, *p* < 0.0001). Although no significant difference in caloric intake was observed between the groups, at Visit 2, the LRD group showed a trend toward higher energy intake compared to the AID group, consistent with their elevated calprotectin levels and the correlation results. Moreover, the marked decrease in caloric intake during cancer treatment may have contributed to the temporary reduction in calprotectin levels observed halfway through CRT. This effect could be attributed to the downregulation of intestinal immune activity amid significant caloric restriction, which temporarily reduces neutrophil infiltration and local inflammatory markers (34). However, sustained energy restriction during treatment, coupled with low nutrient density and fiber intake in the LRD group, may have compromised the integrity of the intestinal barrier and limited mucosal recovery (34). This situation may favor a damaged microenvironment prone to persistent subclinical inflammation, as evidenced by the subsequent increase in calprotectin levels observed at the 3-month post-treatment follow-up.

In contrast, patients in the AID group experienced a more gradual caloric reduction, resulting in stable calprotectin levels throughout treatment and an apparently consistent local inflammatory response. It is important to note that while calprotectin is widely used, it is not the gold standard for assessing gut inflammation. A histological analysis and *in situ* cytokine profiling from intestinal mucosal biopsies would provide more precise and localized information; however, these assessments were not feasible in this study (35).

Dietary compounds, both nutritive and non-nutritive, can have subclinical biological effects related to inflammation. The gut microbiota processes these compounds, producing secondary metabolites that have garnered significant attention in recent research, particularly for their immune-modulatory capabilities. These secondary metabolites can initially modulate the mucosal immune system, leading to a complex series of responses that may result in systemic reactions. Therefore, we assessed systemic inflammation to evaluate the inflammatory or anti-inflammatory effects of the dietary interventions. Our findings indicated that, compared to the AID, levels of IL-1β were elevated in the LRD group at Visit 3, and IL-17A levels were higher at Visit 5. While these findings do not conclusively demonstrate that the AID possesses potent immunosuppressive properties, they suggest a potential immune-modulatory effect that is often expected from non-pharmacological nutritional interventions. It is important to note that any dietary intervention alone may not be sufficient to counteract the pro-inflammatory and oxidative impacts of aggressive cancer treatments such as CRT. Additionally, several uncontrolled variables could have influenced the inflammatory response, including environmental pollution, medications, infections, emotional stress, sleep quality, and other lifestyle factors (36).

In this study, we analyzed the effects of dietary interventions over approximately 5 months, during which patients had the opportunity to make lifestyle changes and develop healthy nutritional habits. We examined the impact of these interventions on treatment response, the occurrence of proctopathy, and OS. The incidence of proctopathy was similar among interventions, as it may be attributed to the extensive damage caused by RT. However, we did observe a trend indicating that a higher proportion of patients in the AID group achieved a complete response and demonstrated longer OS compared to those in the LRD group. These findings underscore the significant value of individualized nutritional care and the use of functional foods to provide the body with the necessary elements for immune defense and self-repair mechanisms, ultimately leading to better treatment responses and more prolonged survival.

This study has several limitations. Physical activity was neither prescribed nor assessed, which may have influenced the outcomes. We did not reach the calculated sample size. The withdrawal rate was higher than anticipated (over 20%), resulting in a smaller final sample size and reduced statistical power. A few patients were excluded because of malnutrition, unmanageable toxicity, and disease progression. The reason for exclusion was toxicity that led to hospitalization and the need for nutritional support; therefore, these patients received an additional intervention different from the dietary arm. The patients excluded for disease progression had progression before CRT began and received a change in treatment. This is a limitation and a bias. Additionally, some patients seemed to follow physicians’ nutritional advice more closely than nutritionists’, which may have affected adherence to the assigned dietary protocols. Unfortunately, the COVID-19 pandemic likely introduced external economic pressures that affected patients’ food choices and their ability to comply with dietary recommendations. Finally, dietary adherence was assessed using a 24-hour dietary recall questionnaire, which has the main limitation of relying on the patient’s memory. Due to resource limitations, implementing an adherence biomarker panel was not feasible; therefore, no objective adherence biomarker was used.

Despite these limitations, to our knowledge, this is the first study comparing different nutritional interventions aimed at reducing the incidence of malnutrition in CC patients undergoing CRT. The study assessed a broad range of clinical outcomes and provided meaningful findings through dietary modifications alone. Future studies should consider incorporating physical activity guidelines to potentially enhance outcomes. Including gut microbiota analysis may also offer valuable insights into the connections between diet, inflammation, treatment response, and overall health.

## 5 Conclusion

This study reinforces the growing body of evidence supporting the role of diet and nutrition as modifiable factors in cancer treatment. Our findings consistently showed that even with the lack of statistically significant differences, the AID tended to nutritional status restoration, early recovery from toxicity symptoms and stability in the inflammatory response, in addition to higher complete disease response rates, and a tendency to prolonged OS. These results suggest that dietary patterns rich in fiber, omega-3 fatty acids, and antioxidants may contribute to improved clinical outcomes during and after CRT. Conversely, while the LRD may help alleviate diarrhea severity, its restrictive nature, especially when food intake is already limited, is not recommended in the long term because the lack of nutrients may lead to nutritional deficiencies.

Given the complex nature of inflammation and cachexia in cancer patients, along with the subtle effects of dietary interventions, diet alone is unlikely to fully mitigate the adverse effects of CRT. Nevertheless, this study provides evidence that dietary modifications can aid in managing these effects when integrated into a comprehensive supportive care framework.

## Data Availability

The raw data supporting the conclusions of this article will be made available by the authors, without undue reservation.
