# Supplementary material for: Benefits of an anti-inflammatory diet compared to a low-residue diet during concurrent chemoradiation therapy for patients with locally advanced cervical cancer: a randomized clinical trial
Source: Front Nutr. 2026 Jul 13;13:1835417. doi: 10.3389/fnut.2026.1835417 (PMC13402472; doi:10.3389/fnut.2026.1835417)
Supplement: Supplementary file 1 [file Data_Sheet_1.zip › Supplementary material S1.docx]

Supplementary Material S1

Nutritional intervention and assessment

***Nutritional recommendations***

Energy and protein requirements for all patients were based on the Mexican Nutritional Consensus. In patients without comorbidities, an energy intake of 28 to 31 kcal per kilogram of body weight per day was recommended. The suggested protein intake for this group was between 1.1 and 1.3 g/kg/day. When patients presented with comorbidities, the diet was individualized. In cases of obesity or diabetes, energy requirements were calculated using the Harris-Benedict formula, applying the patient’s adjusted body weight. In these cases, the recommended protein intake was approximately 1 g/kg/day. In patients with renal insufficiency, energy recommendations varied depending on age. For those aged 60 years and older, an intake of 30 kcal/kg/day was advised, while for patients under 60 years of age, the recommendation was 35 kcal/kg/day. Protein intake also depended on the degree of renal impairment. In cases of chronic kidney disease without replacement therapy and with a glomerular filtration rate (GFR) below 25 ml/min, a protein intake of 0.6 g/kg/day was recommended, with at least two-thirds coming from high biological value proteins. For patients with a GFR between 25 and 70 ml/min, the same recommendation was maintained. In contrast, for those with a GFR greater than 79 ml/min, protein intake could range from 0.8 to 1 g/kg/day. In cases of acute renal failure, energy requirements were calculated using the Harris-Benedict formula with a correction factor of 1.3; protein intake was adjusted to a range of 0.5 to 1 g/kg/day.

In geriatric patients without comorbidities, energy needs were also determined using the Harris-Benedict formula. The recommended protein intake for this group ranged from 0.9 to 1.1 g/kg/day.

Patients in the AID group were indicated 30% of fat from total energy and were advised to increase their intake of anti-inflammatory compounds, soluble fiber, and probiotics. Patients were provided with green tea, chia seeds, ginger, and turmeric to support adherence to the AID. In contrast, those in the LRD group followed a diet low in fiber (≤20 g), lactose (≤5 g), and fat (≤20% of total energy intake).

The trial included five visits: V1, two weeks before CRT; V2, on the day CRT started; V3, after three cycles of CT; V4, after BT; and V5, 12 weeks after BT V5. For both groups, each visit lasted 1 hour and included nutritional counseling and diet-related problem-solving strategies. Three collaborators were included, and participants were provided with a phone number for any diet- or health-related concerns.

Also, visually engaging printed materials were provided as a guide, based on the characteristics and requirements of each dietary intervention, to facilitate adherence to both interventions. For both groups, the dietary guide was adapted from the Mexican Food Equivalents System.

Here is an example of the material provided as a guide for the AID group (in Spanish):


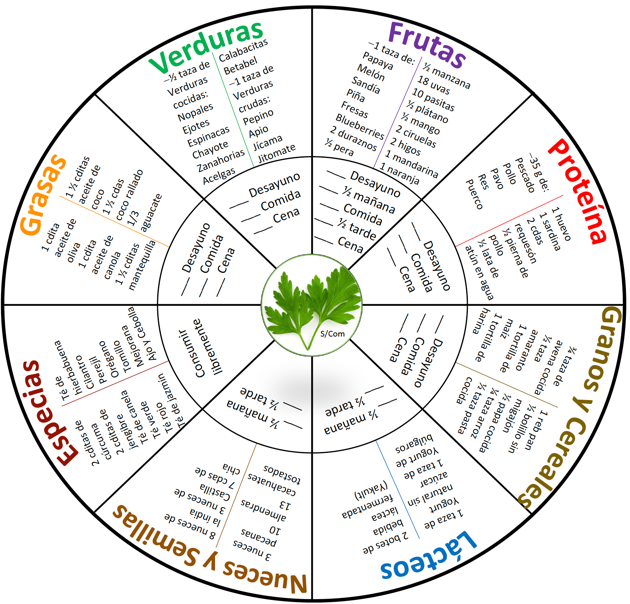

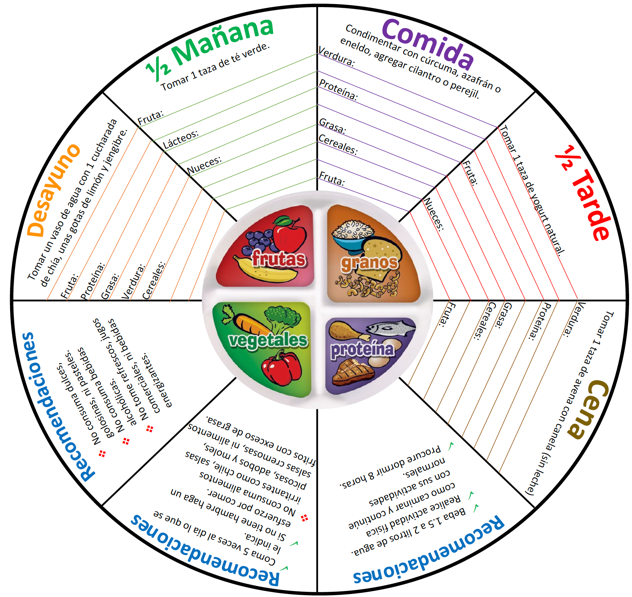


This is the material for the LRD group (in Spanish):


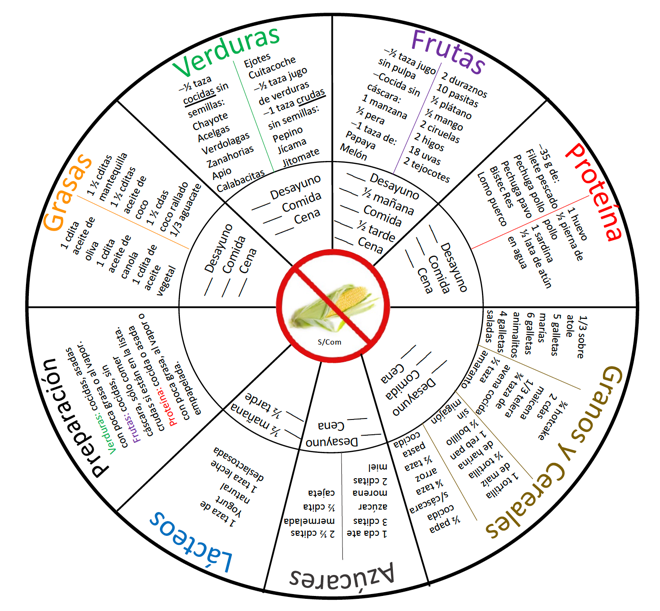

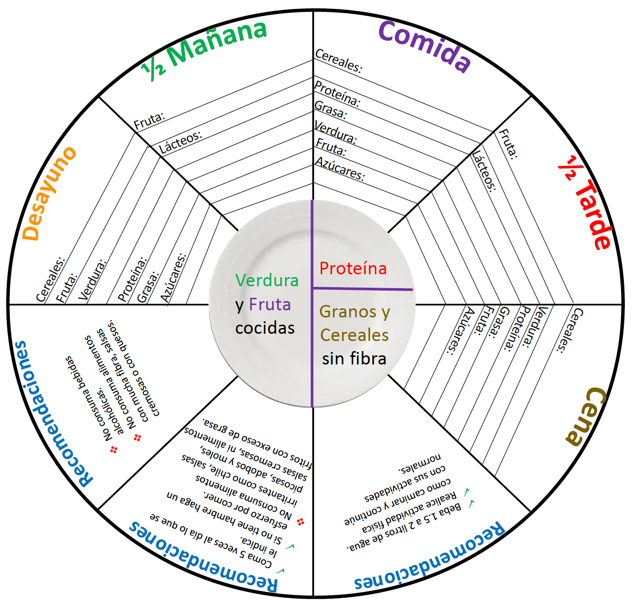


During V1, patients received explanations of their assigned diet, the materials mentioned, and underwent a baseline evaluation.

***Dietary Assessment***

The dietary intake of each patient was assessed using a 24-hour dietary recall during each visit. The Mexican System of Equivalent Foods was employed to quantify energy, protein, fat, and carbohydrates during each evaluation (20). To measure both the nutritive and non-nutritive components of the diet, we used the Food Processor software (version 10.8.0.0).

***Anthropometric measurements***

Weight was measured using a digital flat scale (SECA model 810; SECA Corp., Hamburg, Germany), and height was measured using a portable stadiometer (SECA model 213; SECA Corp., Hamburg, Germany). Body Mass Index (BMI) was calculated using the formula: BMI $=$ weight (kg) $\div$ height (m)^2^.

***Undernutrition assessment***

The Patient-Generated Subjective Global Assessment (PG-SGA) was used to assess nutritional risk (21). A patient was considered malnourished if they met two or more of these criteria: PG-SGA categories B or C, BMI under 18.5 kg/m², significant weight loss, albumin below 3.4 g/dL, hemoglobin below 12 g/dL, and energy intake below 90% of the requirement (22).

***Body composition analysis***

Skeletal Muscle (SM) was assessed from cross-sectional CT images of the third lumbar (L3) vertebra, taken before treatment and three months post-treatment. Two trained researchers used the HOROS software (USA, v. 4.0.1) for analysis. The skeletal muscle area was calculated using Hounsfield Units (HU) between -29 and +150 (23). SM was then normalized to the patient’s squared height (cm²/m²) to derive the skeletal muscle index (SMI). Moderate sarcopenia was identified when SMI was below 38.5 cm²/m² (24). Muscle strength was evaluated through handgrip strength in the dominant hand with a dynamometer (Smedley T-18, Takei Scientific Instruments Co. Ltd., Niigata, Japan).
