# Supplementary material for: Benefits of an anti-inflammatory diet compared to a low-residue diet during concurrent chemoradiation therapy for patients with locally advanced cervical cancer: a randomized clinical trial
Source: Front Nutr. 2026 Jul 13;13:1835417. doi: 10.3389/fnut.2026.1835417 (PMC13402472; doi:10.3389/fnut.2026.1835417)
Supplement: Supplementary file 1 [file Data_Sheet_1.zip › Supplementary material S2.docx]

Supplementary Material S2

Missing Data Sensitivity Analysis

**Percentage and Pattern of Missing Data**

The initial evaluation of the database revealed an overall missing data proportion of 5.2%. However, when analyzing missingness patterns at the univariate level, certain clinical variables of interest exhibited substantially higher omission rates (up to 39% for longitudinal variables such as IL-1Beta). Although the imputation process was applied to the complete dataset, our sensitivity and diagnostic analyses focused exclusively on variables with a missing rate of 5% or greater.


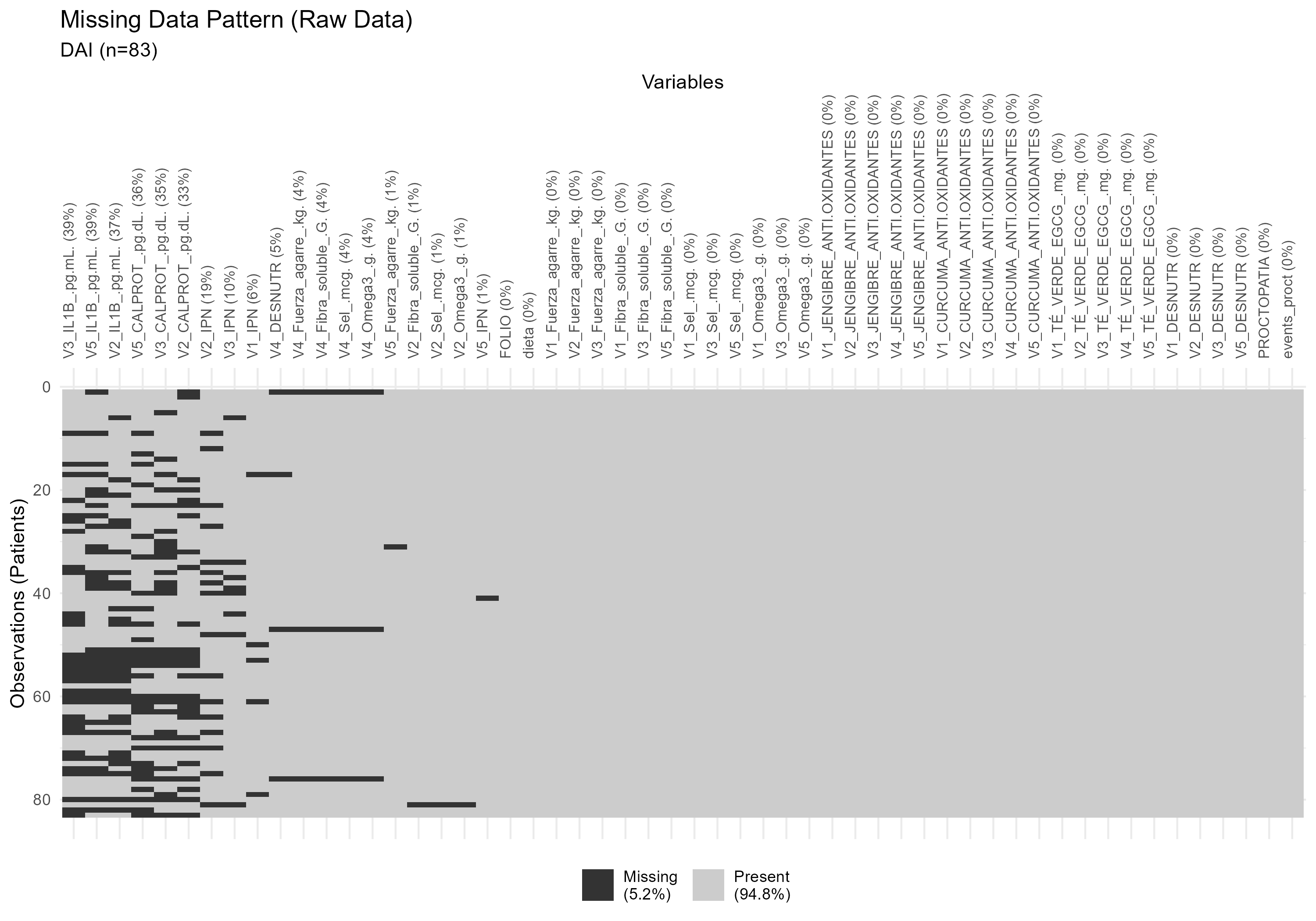


**Imputation Process**

To address the missing data, the Multiple Imputation by Chained Equations (MICE) algorithm was implemented. The Predictive Mean Matching (PMM) method was specifically selected given its semi-parametric nature; this choice avoids assuming distributional normality and guarantees that imputations derive exclusively from real observed values, ensuring the biological plausibility of the results. A total of m = 35 imputed datasets were generated, each with t = 15 iterations, utilizing a pool of k = 10 potential donors.

The predictor matrix for the univariate models within the algorithm was configured based on the analysis of correlograms, Principal Component Analysis (PCA), clinical relevance, and previous longitudinal measurements of the target variable. Furthermore, severe multicollinearity among the regressor variables was assessed and ruled out. The convergence of the Gibbs sampling was visually confirmed by evaluating the stability of the means and variances across iterations in the Markov chains.

**Evaluation of the Imputation**

Analysis of post-imputation diagnostics, including the calculation of balance ratios and donor selection frequencies, confirmed the absence of 'donor fatigue', effectively ruling out the creation of artificial modes in the data.

**Table. Potential donor fatigue**

| Variable | Missing N | Missing % | Shared Donor Value | Observed Count (Before Imputation) | Final Count (After Imputation) | PMM Times  Imputed | Balance Ratio (Observed/Reused) |
| --- | --- | --- | --- | --- | --- | --- | --- |
| V3_IL1B_.pg.mL. | 32 | 38.6 | 3.5 | 4 | 12 | 8 | 0.50 |
|  |  |  | 2.9 | 2 | 9 | 7 | 0.29 |
|  |  |  | 2.7 | 7 | 13 | 6 | 1.17 |
|  |  |  | 3.3 | 5 | 8 | 3 | 1.67 |
|  |  |  | 2.3 | 2 | 4 | 2 | 1.00 |
|  |  |  | 2.6 | 2 | 4 | 2 | 1.00 |
|  |  |  | 2.9 | 2 | 4 | 2 | 1.00 |
|  |  |  | 3.1 | 2 | 4 | 2 | 1.00 |
|  |  |  | 3.2 | 3 | 5 | 2 | 1.50 |
|  |  |  | 3.2 | 3 | 5 | 2 | 1.50 |
|  |  |  | 2.6 | 1 | 2 | 1 | 1.00 |
|  |  |  | 3.6 | 1 | 2 | 1 | 1.00 |
|  |  |  | 2.8 | 2 | 3 | 1 | 2.00 |
|  |  |  | 3.8 | 2 | 3 | 1 | 2.00 |
|  |  |  | 3.0 | 4 | 5 | 1 | 4.00 |
| V5_IL1B_.pg.mL. |  |  | 2.7 | 5 | 10 | 5 | 1.00 |
|  |  |  | 3.2 | 5 | 9 | 4 | 1.25 |
|  |  |  | 4.8 | 1 | 4 | 3 | 0.33 |
|  |  |  | 3.1 | 2 | 5 | 3 | 0.67 |
|  |  |  | 3.3 | 4 | 7 | 3 | 1.33 |
|  |  |  | 3.8 | 4 | 7 | 3 | 1.33 |
|  |  |  | 3.0 | 6 | 9 | 3 | 2.00 |
|  |  |  | 2.3 | 1 | 3 | 2 | 0.50 |
|  |  |  | 2.4 | 2 | 4 | 2 | 1.00 |
|  |  |  | 4.4 | 2 | 4 | 2 | 1.00 |
|  |  |  | 2.9 | 4 | 6 | 2 | 2.00 |
|  |  |  | 2.6 | 2 | 3 | 1 | 2.00 |
|  |  |  | 3.7 | 2 | 3 | 1 | 2.00 |
|  |  |  | 4.0 | 2 | 3 | 1 | 2.00 |
| V2_IL1B_.pg.mL. | 31 | 37.3 | 2.9 | 7 | 15 | 8 | 0.88 |
|  |  |  | 2.6 | 3 | 8 | 5 | 0.60 |
|  |  |  | 2.9 | 2 | 6 | 4 | 0.50 |
|  |  |  | 3.2 | 5 | 9 | 4 | 1.25 |
|  |  |  | 2.8 | 1 | 4 | 3 | 0.33 |
|  |  |  | 3.6 | 1 | 4 | 3 | 0.33 |
|  |  |  | 3.1 | 3 | 6 | 3 | 1.00 |
|  |  |  | 3.3 | 4 | 7 | 3 | 1.33 |
|  |  |  | 2.7 | 2 | 4 | 2 | 1.00 |
|  |  |  | 2.5 | 1 | 2 | 1 | 1.00 |
|  |  |  | 3.7 | 1 | 2 | 1 | 1.00 |
|  |  |  | 4.5 | 1 | 2 | 1 | 1.00 |
|  |  |  | 3.5 | 4 | 5 | 1 | 4.00 |
| V5_CALPROT_.pg.dL. | 30 | 36.1 | 0.0 | 15 | 21 | 6 | 2.50 |
|  |  |  | 1,251.3 | 1 | 6 | 5 | 0.20 |
|  |  |  | 413.2 | 1 | 5 | 4 | 0.25 |
|  |  |  | 111.6 | 1 | 4 | 3 | 0.33 |
|  |  |  | 562.2 | 1 | 4 | 3 | 0.33 |
|  |  |  | 445.6 | 1 | 3 | 2 | 0.50 |
|  |  |  | 663.5 | 1 | 3 | 2 | 0.50 |
|  |  |  | 725.4 | 1 | 3 | 2 | 0.50 |
|  |  |  | 884.9 | 1 | 3 | 2 | 0.50 |
|  |  |  | 2,337.2 | 1 | 3 | 2 | 0.50 |
|  |  |  | 212.9 | 1 | 2 | 1 | 1.00 |
|  |  |  | 228.7 | 1 | 2 | 1 | 1.00 |
|  |  |  | 299.8 | 1 | 2 | 1 | 1.00 |
|  |  |  | 593.0 | 1 | 2 | 1 | 1.00 |
|  |  |  | 661.1 | 1 | 2 | 1 | 1.00 |
|  |  |  | 686.9 | 1 | 2 | 1 | 1.00 |
|  |  |  | 720.4 | 1 | 2 | 1 | 1.00 |
|  |  |  | 765.2 | 1 | 2 | 1 | 1.00 |
|  |  |  | 1,101.2 | 1 | 2 | 1 | 1.00 |
|  |  |  | 1,334.9 | 1 | 2 | 1 | 1.00 |
| V3_CALPROT_.pg.dL. | 29 | 34.9 | 0.0 | 22 | 27 | 5 | 4.40 |
|  |  |  | 312.7 | 1 | 4 | 3 | 0.33 |
|  |  |  | 957.2 | 1 | 4 | 3 | 0.33 |
|  |  |  | 49.0 | 1 | 3 | 2 | 0.50 |
|  |  |  | 329.3 | 1 | 3 | 2 | 0.50 |
|  |  |  | 669.2 | 1 | 3 | 2 | 0.50 |
|  |  |  | 972.5 | 1 | 3 | 2 | 0.50 |
|  |  |  | 1,075.3 | 1 | 3 | 2 | 0.50 |
|  |  |  | 33.7 | 1 | 2 | 1 | 1.00 |
|  |  |  | 78.1 | 1 | 2 | 1 | 1.00 |
|  |  |  | 665.8 | 1 | 2 | 1 | 1.00 |
|  |  |  | 892.2 | 1 | 2 | 1 | 1.00 |
|  |  |  | 895.8 | 1 | 2 | 1 | 1.00 |
|  |  |  | 1,004.1 | 1 | 2 | 1 | 1.00 |
|  |  |  | 1,182.0 | 1 | 2 | 1 | 1.00 |
| V2_CALPROT_.pg.dL. | 27 | 32.5 | 1,655.3 | 1 | 5 | 4 | 0.25 |
|  |  |  | 597.1 | 1 | 4 | 3 | 0.33 |
|  |  |  | 566.6 | 1 | 3 | 2 | 0.50 |
|  |  |  | 709.4 | 1 | 3 | 2 | 0.50 |
|  |  |  | 925.4 | 1 | 3 | 2 | 0.50 |
|  |  |  | 1,076.5 | 1 | 3 | 2 | 0.50 |
|  |  |  | 1,087.9 | 1 | 3 | 2 | 0.50 |
|  |  |  | 226.8 | 1 | 2 | 1 | 1.00 |
|  |  |  | 317.1 | 1 | 2 | 1 | 1.00 |
|  |  |  | 327.0 | 1 | 2 | 1 | 1.00 |
|  |  |  | 331.7 | 1 | 2 | 1 | 1.00 |
|  |  |  | 529.2 | 1 | 2 | 1 | 1.00 |
|  |  |  | 563.5 | 1 | 2 | 1 | 1.00 |
|  |  |  | 576.7 | 1 | 2 | 1 | 1.00 |
|  |  |  | 792.6 | 1 | 2 | 1 | 1.00 |
|  |  |  | 819.1 | 1 | 2 | 1 | 1.00 |
|  |  |  | 846.3 | 1 | 2 | 1 | 1.00 |
|  |  |  | 926.2 | 1 | 2 | 1 | 1.00 |
|  |  |  | 1,052.3 | 1 | 2 | 1 | 1.00 |
|  |  |  | 1,088.7 | 1 | 2 | 1 | 1.00 |
|  |  |  | 1,337.5 | 1 | 2 | 1 | 1.00 |
|  |  |  | 1,344.3 | 1 | 2 | 1 | 1.00 |
|  |  |  | 3,347.7 | 1 | 2 | 1 | 1.00 |
|  |  |  | 0.0 | 9 | 10 | 1 | 9.00 |
| V2_IPN | 16 | 19.3 | 37.0 | 1 | 2 | 1 | 1.00 |
|  |  |  | 43.0 | 1 | 2 | 1 | 1.00 |
|  |  |  | 40.0 | 2 | 3 | 1 | 2.00 |
|  |  |  | 41.0 | 2 | 3 | 1 | 2.00 |
| V3_IPN | 8 | 9.6 | 40.0 | 3 | 5 | 2 | 1.50 |
|  |  |  | 42.0 | 1 | 2 | 1 | 1.00 |
|  |  |  | 38.0 | 3 | 4 | 1 | 3.00 |
|  |  |  | 40.0 | 6 | 7 | 1 | 6.00 |
| V1_IPN | 5 | 6.0 | 42.0 | 2 | 3 | 1 | 2.00 |
| V4_DESNUTR | 4 | 4.8 | 0.0 | 63 | 66 | 3 | 21.00 |
|  |  |  | 1.0 | 16 | 17 | 1 | 16.00 |
| V4_Fibra_soluble_.G. | 3 | 3.6 | 1.5 | 1 | 2 | 1 | 1.00 |
|  |  |  | 2.7 | 1 | 2 | 1 | 1.00 |
|  |  |  | 3.4 | 2 | 3 | 1 | 2.00 |
| V4_Fuerza_agarre_.kg. |  |  | 14.0 | 2 | 3 | 1 | 2.00 |
|  |  |  | 20.5 | 2 | 3 | 1 | 2.00 |
|  |  |  | 25.5 | 2 | 3 | 1 | 2.00 |
| V4_Omega3_.g. |  |  | 0.6 | 1 | 2 | 1 | 1.00 |
|  |  |  | 0.8 | 1 | 2 | 1 | 1.00 |
|  |  |  | 0.1 | 2 | 3 | 1 | 2.00 |
| V4_Sel_.mcg. |  |  | 29.8 | 1 | 2 | 1 | 1.00 |
| V2_Omega3_.g. | 1 | 1.2 | 1.2 | 1 | 2 | 1 | 1.00 |
| V2_Sel_.mcg. |  |  | 105.3 | 1 | 2 | 1 | 1.00 |
| V5_Fuerza_agarre_.kg. |  |  | 18.5 | 2 | 3 | 1 | 2.00 |

This table assesses potential 'donor fatigue' during the Predictive Mean Matching (PMM) process for key variables in the analytical cohort (n=83). **Missing N/%** indicates the original amount of missing data per biomarker visit. **Shared 'Donor' Value** represents a specific observed value from a single patient (donor) that was assigned to other patients (receptors) who had missing data. **Observed Count (Before Imputation)** is how many times that specific value was naturally observed in the raw data. **PMM Times Imputed** is how many receptor patients received that specific donor value. **Data Balance Ratio** (Observed Count / Reuse Frequency) assesses the robustness of the matching; ratios greater than 0.2 (e.g., 1 donor shared with 5 receptors max) for extreme values in skewed distributions (like fecal Calprotectin V2, V3, V5) confirm a healthy preservation of empirical variance without over-reliance on single donors.

**Marginal Densities and Longitudinal Consistency**

To evaluate the quality of the imputations for variables with >5% missingness, the marginal densities of the observed data were compared with those of the imputed data. The overlap demonstrates that the distributions' morphology (mean, variance, skewness, and kurtosis) was preserved without significant alteration, ruling out bias toward the mean or artificial inflation of the variance.

Notably, in markers such as Calprotectin (A, C, I), PNI (E, F, G), and IL-1Beta (B, D, J), the PMM method successfully respected the intrinsic positive skewness of the clinical variables. The consistency of these densities across the different time points suggests that the model adequately captured the intra-subject longitudinal covariance structure.

**
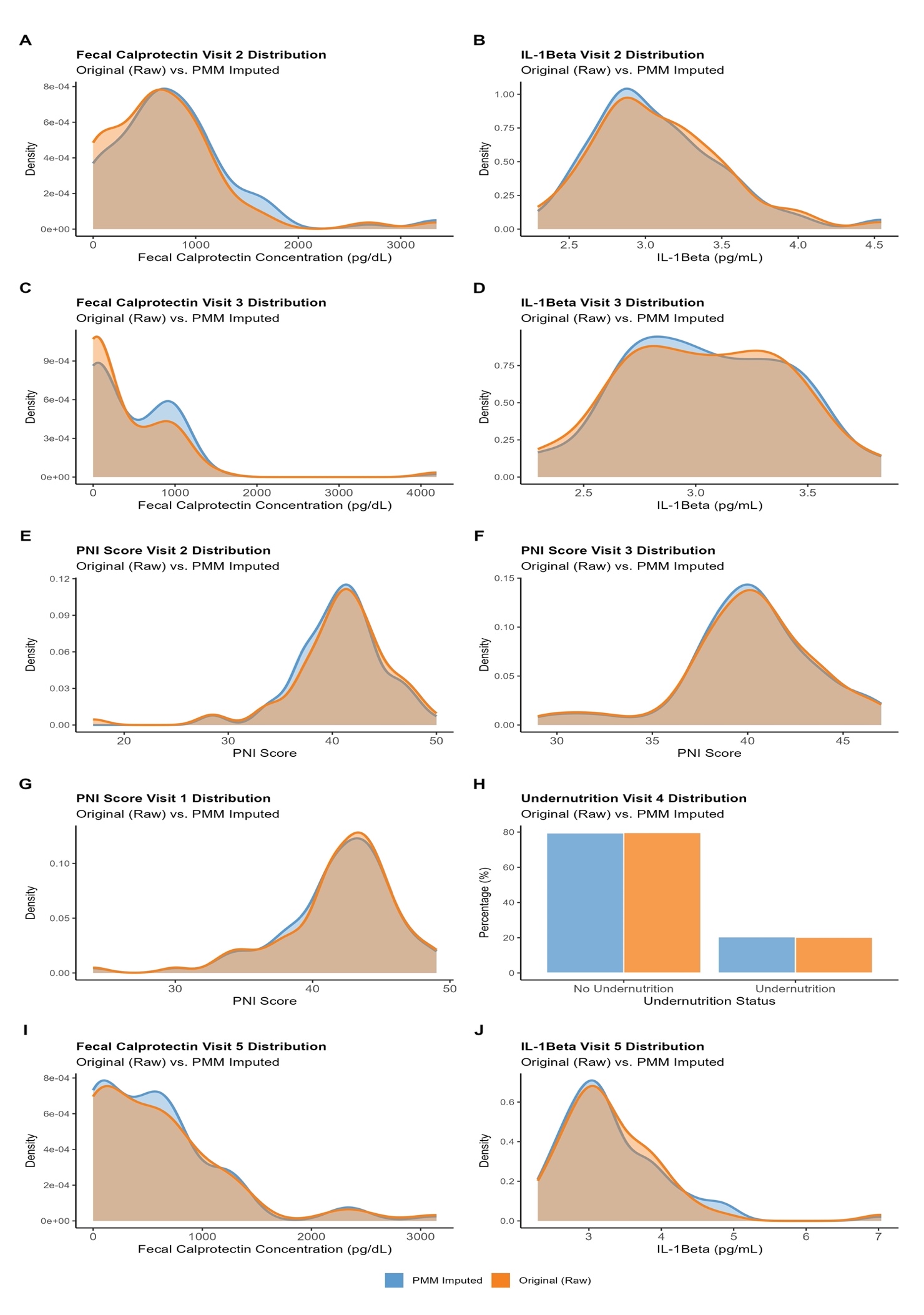
**

**Sensitivity Analysis (Complete-Case vs. Imputed). The following figure shows a comparison between imputed dietary data versus raw data.**

Our analysis utilizes Fixed Effects Models to better capture the longitudinal dynamics of nutritional interventions. Alongside this update, we performed the complete-case sensitivity analysis and provided the non-imputed clinical trajectories for visual comparison. However, from a statistical standpoint, relying on Complete-Case Analysis (CCA) as the primary inferential tool introduces severe methodological limitations across our dual statistical framework. The missing data pattern observed in our study is characteristic of longitudinal attrition.

Consequently:

- **For longitudinal assessments (Friedman test):** The strict listwise deletion inherent to non-parametric repeated-measures tests discards entire patient records even if 90% to 95% of their variables were successfully recorded across other visits.
- **For cross-sectional group comparisons (Mann-Whitney U test):** Pairwise deletion at specific time points with high missingness (e.g., reaching up to 39% at Visit 3) drastically reduces the effective sample size for those specific evaluations.

Taken together, this systematic loss of data severely deflates the effective sample size (N), massively inflates variance, and mathematically increases the probability of Type II errors.

Furthermore, this longitudinal dropout pattern empirically violates the Missing Completely At Random (MCAR) assumption required for an unbiased CCA. While a Missing Not At Random (MNAR) mechanism can never be definitively ruled out, this structural reality strongly aligns with a Missing At Random (MAR) mechanism, validating our selection of the MICE framework to appropriately model longitudinal covariance and recover statistical power.

To verify that our multiple imputation process did not introduce selection bias or artificial significance, we conducted a trajectory analysis comparing the PMM-imputed datasets against the raw CCA data, alongside post-imputation diagnostic checks:

1. **Structural Isomorphism and Directional Consistency:** As the Supplementary Figures demonstrate, the structural isomorphism between the imputed and raw trajectories is remarkably consistent. The CCA reveals expected shifts in scale (e.g., Beta-Carotene) and high volatility in group trajectories (e.g., Lycopene) compared to the imputed dataset. This volatility illustrates the massive inflation of standard errors caused by the missing data. The PMM algorithm successfully stabilized these trajectories—handling extreme values through real-world donor matching—and faithfully preserved the natural biological trends of the cohort without artificially distorting the group means.
2. **Detrimental Impact of Listwise Deletion on Valid Data:** The complete-case analysis visually demonstrates the negative consequences of listwise deletion in longitudinal datasets. As observed in the previous Figures for dietary variables (e.g., Total Fiber, Ginger AOx, Lutein-Zeaxanthin, Turmeric, Green Tea, Magnesium, and Vitamin A), the complete-case approach discards entire patient records due to missingness in unrelated variables or at isolated time points. This unnecessary exclusion of perfectly valid observed data arbitrarily shifts the group means and severely depletes statistical power, leading to a loss of significance. By employing MICE, we successfully retained these valid partial trajectories, maximizing the use of observed data and providing a much more accurate and statistically robust estimation of the true dietary trends.
3. **Distributional Integrity and Absence of Synthetic Artifacts:** The ultimate proof of our algorithm's neutrality lies in our post-imputation diagnostics. The marginal densities of the imputed values perfectly overlapped with the observed raw data, accurately preserving the original variance, kurtosis, and biological skewness (e.g., Calprotectin, IL-1Beta, PNI). This proves that the PMM method did not force values toward an artificial mean or inject synthetic trends to favor our hypothesis, but strictly drew from the existing physiological space of the observed donors.

In conclusion, while the CCA naturally suffers from attenuated p-values due to a severely reduced sample size (N), the clinical trajectories and effect directionality between the AID and LRD groups remain biologically consistent. This confirms that our MICE approach successfully recovered the necessary statistical power to detect true clinical trends without distorting the underlying clinical reality.
